# Stabilization of CXCL12 (SDF-1α) via silk fibroin films enhances stem cell migration/retention and functional recovery after stroke

**DOI:** 10.1093/rb/rbaf129

**Published:** 2025-12-12

**Authors:** Amira Lekouaghet, Marta Sánchez-Díez, José Pérez-Rigueiro, Francisco J Rojo, Carmen Ramírez-Castillejo, Yolanda Ruiz-León, Fivos Panetsos, Gustavo V Guinea, Daniel González-Nieto

**Affiliations:** Center for Biomedical Technology, Universidad Politécnica de Madrid, Pozuelo de Alarcón 28223, Spain; Center for Biomedical Technology, Universidad Politécnica de Madrid, Pozuelo de Alarcón 28223, Spain; Center for Biomedical Technology, Universidad Politécnica de Madrid, Pozuelo de Alarcón 28223, Spain; Departamento de Ciencia de Materiales, ETSI Caminos, Universidad Politécnica de Madrid, Madrid 28040, Spain; Centro de Investigación Biomédica en Red de Bioingeniería, Biomateriales y Nanomedicina (CIBER-BBN), Instituto de Salud Carlos III, Madrid 28029, Spain; Biomaterials and Regenerative Medicine Group, Instituto de Investigación Sanitaria del Hospital Clínico San Carlos (IdISSC), Madrid 28040, Spain; Silk Biomed SL, Galapagar 28260, Spain; Center for Biomedical Technology, Universidad Politécnica de Madrid, Pozuelo de Alarcón 28223, Spain; Departamento de Ciencia de Materiales, ETSI Caminos, Universidad Politécnica de Madrid, Madrid 28040, Spain; Centro de Investigación Biomédica en Red de Bioingeniería, Biomateriales y Nanomedicina (CIBER-BBN), Instituto de Salud Carlos III, Madrid 28029, Spain; Biomaterials and Regenerative Medicine Group, Instituto de Investigación Sanitaria del Hospital Clínico San Carlos (IdISSC), Madrid 28040, Spain; Silk Biomed SL, Galapagar 28260, Spain; Center for Biomedical Technology, Universidad Politécnica de Madrid, Pozuelo de Alarcón 28223, Spain; Research Support Unit, Real Jardín Botánico, Consejo Superior de Investigaciones Científicas (CSIC), Madrid 28014, Spain; Biomaterials and Regenerative Medicine Group, Instituto de Investigación Sanitaria del Hospital Clínico San Carlos (IdISSC), Madrid 28040, Spain; Silk Biomed SL, Galapagar 28260, Spain; Neurocomputing and Neurorobotics Research Group, Faculty of Biology and Faculty of Optics, Universidad Complutense de Madrid, Madrid 28040, Spain; Center for Biomedical Technology, Universidad Politécnica de Madrid, Pozuelo de Alarcón 28223, Spain; Departamento de Ciencia de Materiales, ETSI Caminos, Universidad Politécnica de Madrid, Madrid 28040, Spain; Centro de Investigación Biomédica en Red de Bioingeniería, Biomateriales y Nanomedicina (CIBER-BBN), Instituto de Salud Carlos III, Madrid 28029, Spain; Biomaterials and Regenerative Medicine Group, Instituto de Investigación Sanitaria del Hospital Clínico San Carlos (IdISSC), Madrid 28040, Spain; Silk Biomed SL, Galapagar 28260, Spain; Center for Biomedical Technology, Universidad Politécnica de Madrid, Pozuelo de Alarcón 28223, Spain; Centro de Investigación Biomédica en Red de Bioingeniería, Biomateriales y Nanomedicina (CIBER-BBN), Instituto de Salud Carlos III, Madrid 28029, Spain; Silk Biomed SL, Galapagar 28260, Spain; Departamento de Tecnología Fotónica y Bioingeniería, ETSI Telecomunicaciones, Universidad Politécnica de Madrid, Madrid 28040, Spain

**Keywords:** brain stroke, silk fibroin films, SDF-1α, neuroprotection

## Abstract

Brain pathologies such as ischemic stroke or traumatic brain injury (TBI) are among the most impactful diseases worldwide. In ischemic stroke, we currently lack truly effective treatments capable of delaying infarct progression, limiting lesion size or stimulating endogenous brain repair mechanisms to promote neurovascular remodeling and functional recovery. Two main barriers continue to limit the clinical translation of therapeutic molecules: the highly restrictive nature of the blood–brain barrier and that many bioactive molecules exhibit low stability at the target site, with half-lives shorter than the therapeutic window. In this study, we developed tunable silk fibroin (SF) films of variable concentration, fabricated via water annealing, that effectively preserve the functional activity of the chemokine CXCL12 (SDF-1α). The 2% SF formulation provided sustained release of SDF-1α for at least 7 days, promoting the *in vitro* migration of mesenchymal stem cells (MSCs) and low-density bone marrow mononuclear cells (LDBM), the latter containing hematopoietic stem cells. When implanted on the cortical surface, the SDF-1α-SF films successfully stimulated the guided migration of exogenously administered MSCs and LDBM from subcortical regions into the cerebral cortex. Furthermore, co-implantation of SDF-1α-SF films with MSCs or LDBM enhanced cell retention at the cortical site, effectively minimizing off-target dispersion. In a photothrombotic model of cortical ischemia, allowing precise control of lesion location and size, SDF-1α-SF films significantly reduced lesion volume and preserved neuronal function in the somatosensory cortex, as assessed by electrophysiology. Our findings provide proof of concept for using chemokine-releasing biomaterials to actively modulate stem cell migration and retention within the brain, offering strong potential for neuroprotection and tissue remodeling in areas at risk or already affected by damage.

## Graphical Abstract

**Figure:**
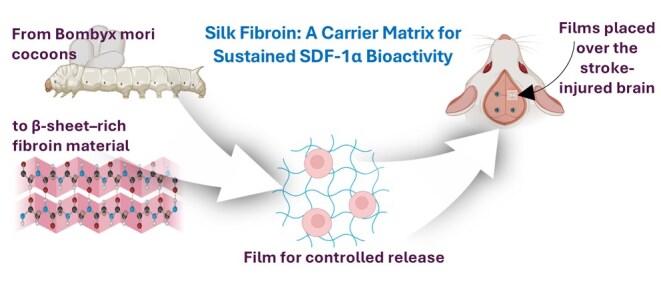

## Introduction

Brain stroke is the second most frequent cause of death and a main leading cause of adult disability [1]. Despite advances in acute management, such as intravenous thrombolysis, endovascular therapy, and specialized stroke unit care, only a small fraction of patients (<10%) receive thrombolytic treatment [2], and many remain ineligible for or unresponsive to current interventions. It is mandatory to identify realistic therapies to manage acute (<24 h) and subacute stroke phases (∼5–7 days), two clinical time periods when the brain probably retains its greatest potential for functional recovery.

The clinical efficacy of cell-based therapies for stroke remains controversial, as human trials have yielded inconsistent and inconclusive outcomes [3]. At both preclinical and clinical levels, most cell-based therapies rely on the intravenous or localized (i.e. intracranial) transplantation of mesenchymal stem cells (MSCs) or hematopoietic stem cells (HSCs) and progenitors. In parallel, pluripotent stem cells, either embryonic or induced pluripotent stem cells, have also been investigated. However, a persistent challenge lies in directing these cells efficiently to injured brain regions, a prerequisite for effective tissue repair.

Stem cell migration is governed by complex chemotactic signaling that becomes particularly active under inflammatory or injury [4, 5]. Although it has long been known that endogenous HSCs exhibit bidirectional movement between the bone marrow and the bloodstream under physiological conditions [5, 6], their potential to home to distant non-hematopoietic tissues such as the brain is extremely rare and remains poorly understood. By contrast, MSCs are rarely present in peripheral blood but become significantly more abundant after tissue injury, such as trauma, cardiac dysfunction, liver damage, or cancer. Current challenges focus on detecting these cells accurately and defining their functional, phenotypic, and molecular characteristics [7]. Importantly, *ex vivo* manipulation, such as prolonged culture or expansion, can alter the natural trafficking of stem cells and reduce therapeutic efficacy [8]. This disruption of their natural trafficking mechanisms may represent a major limitation of the success of clinical transplantation strategies, further emphasizing the need to design strategies that preserve or restore stem cell migratory behavior.

A variety of chemotactic factors have demonstrated considerable potential in orchestrating stem cell trafficking. Among them, monocyte chemoattractant protein-1 (MCP-1) has been shown to promote the migration of neural progenitors and MSCs through its interaction with the CCR2 receptor, thereby facilitating efficient homing to sites of tissue injury [9, 10]. Similarly, the stromal cell-derived factor-1 (SDF-1α/CXCL12)–CXCR4/CXCR7 axis plays a central and well-characterized role in stem cell guidance and retention, regulating the migration not only of HSCs but also of neural stem/progenitor cells, particularly in the context of ischemic brain injury [11, 12]. However, these molecules are highly unstable, with half-lives of only a few hours—much shorter than the therapeutic window required for meaningful biological effects. The incorporation of these factors into biomaterial-based delivery systems offers a promising approach to enhance their stability and sustained bioactivity.

Biomaterials such as chitosan, hyaluronic acid, laminin, collagen, silk fibroin (SF), methylcellulose, polyethylene glycol, polylactic acid, thermoresponsive polymers, and Matrigel have been extensively used in drug delivery and tissue repair [13, 14]. From a clinical standpoint, the therapeutic benefits observed in most biomaterial-based preclinical studies are often offset by concerns regarding the invasiveness of implantation procedures. Among these materials, SF stands out for its outstanding biocompatibility, minimal immunogenicity, mechanical robustness, and intrinsic ability to self-assemble [15]. Our previous work demonstrated that SF is well tolerated in the central nervous system (CNS), eliciting only mild inflammatory responses [16]. Moreover, SF exhibits remarkable stability and slow degradation even under pronounced neuroinflammatory conditions such as stroke or neurodegenerative disease [17].

In earlier studies, we showed that SF hydrogels can serve as delivery platforms for bioactive molecules with antioxidant, anti-inflammatory, and pro-angiogenic properties [18]. Nonetheless, SDF-1α-loaded SF hydrogels failed to promote efficient release or induce measurable cell migration responses, thereby limiting their chemotactic potential.

To overcome these limitations, in this study, we engineered an implantable SF film designed to encapsulate chemotactic molecules and allow epicortical application with minimal invasiveness. This scaffold promoted both the *in vitro* and *in vivo* chemotaxis and retention of MSCs and hematopoietic mononuclear cells, a population that contains a small fraction of HSCs. When implanted on the cortical surface, this SF-based construct significantly reduced cerebral damage and preserved the functional activity of the somatosensory cortex following injury, as demonstrated by electrophysiological assessments. Together, these findings highlight the potential of SF films to deliver therapeutic molecules, including chemotactic factors, to facilitate the recruitment of stem cell populations to sites of tissue or organ injury.

## Materials and methods

### SF extraction, purification and film preparation

SF was extracted and purified from *Bombyx mori* cocoons according to the following protocol [19]. Cocoons were generously provided by Dr Salvador D. Aznar-Cervantes (Instituto Murciano de Investigación y Desarrollo Agrario y Medioambiental-IMIDA, Murcia, Spain) and were initially cut into small pieces and degummed to remove sericin in sodium carbonate solution at 0.2% (w/v), for 30 min at 100°C. After degumming, the resulting fibroin fibers were repeatedly rinsed with distilled water and left to dry overnight at room temperature. Dried fibers were then dissolved in 9.4 M lithium bromide (LiBr; Thermo Fisher Scientific) aqueous solution for 4 h at 60°C. The obtained solution was dialyzed against distilled water to remove lithium bromide traces. A total of six dialysis water changes were carried out over 48 h, until the electrical conductivity dropped below 10 μS/cm. The dialyzed solution was centrifuged to eliminate residual impurities and stored at 4°C until use.

Prior to film fabrication, the SF solution was sterilized by filtration through a 0.22-µm membrane filter, a method selected to preserve the native molecular weight distribution and mechanical properties, which can be compromised by autoclaving [20]. The concentration of the sterile SF dope was then determined by drying an aliquot to constant weight. All subsequent casting, drying, and packaging steps were performed under aseptic conditions within a Class II biological safety cabinet using sterile, single-use materials.

SF films (2–6% v/v) were cast either into 96-well plates for release assays or onto plate lids for film recovery (*in vivo* studies), dispensing 100-µL per film to achieve ∼100-µm thickness. To enhance water stability, various crosslinking approaches were tested, including chemical methods such as methanol treatment [21] and physical methods like autoclaving [22]. Ultimately, water annealing (WA) was selected: films were placed in a vacuum desiccator saturated with water vapor for 1 h, inducing conformational changes in the fibroin structure that rendered the films water-insoluble and mechanically stable for subsequent applications.

### SF films microstructural characterization

The microstructural characterization of SF films was conducted by analyzing their secondary structure distribution using Attenuated Total Reflectance Fourier Transform Infrared Spectroscopy (ATR-FTIR) with Nicolet iS5 FTIR equipped with the iD5 ATR accessory. FTIR spectra of the films, both after evaporation and after insolubilization, were recorded using a clean diamond crystal as background reference. Spectra were obtained by averaging 32 scans over 550–4000 cm^−1^ at 4 cm^−1^ resolution. Amide I peaks were baseline-corrected, and the second derivative was used to determine the initial fitting positions for nine Gaussian functions (Full Width at Half Maximum of 8 cm^−1^), corresponding to distinct secondary structures (Table 1). These band assignments were selected from previous studies [23, 24]. A second band for β-turns was also included to prevent significant peak shifts during the fitting process.

**Table 1:** FTIR peak assignments in the Amide I region for protein secondary structure analysis.

Peak	Wavenumber (cm^−1^)	Assignment
01	1594–1609	Tyr side chain; aggregated strand
02	1610–1620	Aggregated β-strand; intermolecular β-sheet
03	1621–1627	Intermolecular β-sheet
04	1628–1637	Intermolecular β-sheet
05	1638–1655	Random coil
06	1656–1662	Helical structures
07	1663–1670	β-turn
08	1671–1694	β-turn
09	1696–1703	Intermolecular β-sheet

### SF films mechanical characterization

The mechanical properties of SF films were assessed via uniaxial tensile tests with specimens (∼25 × 5 mm) submerged in distilled water at 37 °C. The initial cross-sectional area (Ai) of each specimen was calculated according as Ai=thickness×wide. Samples were mounted vertically in an Instron 5543A machine with a 10-mm gauge length, and the upper end was displaced at 3 mm/min while the lower end remained fixed. Archimedean forces were measured prior to testing. Stress–strain curves were derived from the tensile force (*F*) and displacement (Δ*H*) data recorded by the Instron 5543A, with net force corrected for Archimedean effects. Strain (ε) was calculated as $\varepsilon =\frac{\Delta H}{H_{0}}$, where *H*_0_ is the initial sample height. Stress (σ) was calculated as $\sigma =\frac{F}{A}$, where A is the cross-sectional area of the specimen. The initial Young’s modulus (*E*_i_), as well as the strain and stress at break (εb, σb), was determined from the linear region of the stress–strain curve prior to rupture.

### Scanning electron microscopy

Films of different concentrations (2%, 4% and 6%) were sputter-coated with a thin gold layer using a Q150R S Plus coater (Quorum), and surface images were acquired using a Hitachi S-3000N scanning electron microscope operated at 10 kV.

### SF films release properties

The release of SDF-1α and acetylcholine (ACh) from 2%, 4% and 6% SF films was evaluated over a 7-day period. For stability testing, 10 ng of each molecule was incubated in Phosphate-buffered saline (PBS) at 37°C for either 24 h or 7 days. For release studies, 100-µL SF depots were cast, each containing a total of 10 ng of SDF-1α or ACh. The medium was collected at 1 and 7 days, stored at −80 °C, and concentrations quantified by ELISA (R&D Systems, Cat. MCX120 for SDF-1α; Abcam, Cat. ab65345 for ACh).

### *In vivo* studies

All animal procedures were conducted in accordance with national ethical and legal regulations and were approved by the Ethics Committee of the Universidad Politécnica de Madrid and the Regional Government of Madrid (authorization code PROEX 109.1/20). Adult male CD-1 outbred mice (Charles River Laboratories; RRID: MGI: 5649524), aged 3–4 months and weighing 35–42 g, were used in this study. Animals were housed in standard polycarbonate cages (267 × 208 mm) with *ad libitum* access to water and a maintenance diet (Altromin 1328, Hybrid pellet). Cellulose bedding (Coastline Global) and cotton-based materials were provided for environmental enrichment. The animal facility was maintained at 21 ± 2°C, 40–60% relative humidity, with a 12 h light/dark cycle. Animal welfare was monitored regularly by licensed veterinarians and trained personnel at the Center for Biomedical Technology (Pozuelo de Alarcón, Spain) under blinded conditions. The health status of the animals was confirmed to be free of infectious agents based on routine serological and polymerase chain reaction screening, following FELASA guidelines.

#### Assessment of biological functionality of SDF-1α on LDBM and MSCs migration and retention

These studies evaluated the migration and retention of DiI-labeled MSCs and low-density bone marrow mononuclear cell (LDBM) in response to an SDF-1α gradient toward the cortex. Adult male CD-1 mice were anesthetized with 2% isoflurane and secured in a stereotaxic frame, with eyes protected using petroleum jelly and body temperature maintained at 37 °C. Under aseptic conditions, a burr hole was drilled in the right hemisphere at posterior +0 mm, lateral +2 mm from bregma. For migration studies, 5 × 10^4^ cells/µL were injected using a Hamilton syringe into the striatum at 3-mm depth; for retention studies, 10^4^ cells/µL were injected into the cortex at 1-mm depth. Injection volume was 1 µL at 0.2 µL/min, with the syringe left in place for 5 min to allow diffusion.

The absolute dose of SDF-1α was standardized to 10 ng across all *in vivo* readouts. In both migration and retention studies, mice were randomly assigned to three groups: (1) cortical injection of 1 µL PBS, (2) cortical injection of 1 µL SDF-1α (10^4^ ng/mL stock), or (3) epicortical application of a 2% SF sterile film containing 10 ng SDF-1α (prepared by casting 100 µL depots consisting of 10 µL of a 1000 ng/mL working stock of SDF-1α, mixed with 90 µL diluent).

For the migration study, mice were sacrificed at 24 h or 7 days post-injection, and for the retention study, at 7 days. After transcardial perfusion with cold PBS (pH 7.4) and 4% paraformaldehyde, brains were post-fixed for 1 week and cryoprotected in 30% sucrose. Coronal sections (30 µm) were cut on a freezing microtome at −20 °C, stained with 4',6-diamidino-2-phenylindole (DAPI), and imaged by fluorescence microscopy.

Migration and retention analyses were performed using the Fiji (ImageJ) software with the MorphoLibJ plugin. The DiI (red) channel was converted into a binary mask (cells = 255; background = 0). For each DiI^+^ cluster, MorphoLibJ computed the inertia ellipse from second-order central moments to obtain the major (*a*) and minor (*b*) axes. The elongation index (EI) was then automatically calculated as EI = *a*/*b* (EI = 1: round; EI > 1: increasingly elongated) and used as a morphological proxy for migratory spread.

To minimize selection and observer bias, randomization and blinding were rigorously applied throughout the study. The randomization sequence was generated by an independent lab member using an online randomizer (https://randomizer.org/), and treatment codes were concealed until the end of all analyses. Animals, samples, and data files were identified only by alphanumeric codes. All personnel involved in surgeries and histology remained blinded to treatment identity, and randomized run orders were always used to avoid batch or order effects.

#### Photothrombotic model

Photothrombotic (PT) stroke was induced by intraperitoneal injection of Rose Bengal (Sigma-Aldrich, Cat. 198250-5G; 150 mg/kg, 15 mg/mL in PBS) in fully anesthetized mice. Animals were positioned in a stereotaxic apparatus (David Kopf Instruments), and a midline vertical incision was made to expose the skull. Five minutes post-injection, a 532-nm laser (50 mW, Newwish Optoelectronics) was focused on a 2 mm^2^ area of the right cortex (anteroposterior −1.0 mm, mediolateral +2.5 mm from bregma) for 15 min.

#### Electrodes implantation and recordings of somatosensory evoked potentials

For electrode implantation, animals were anesthetized with isoflurane, and the absence of the pedal withdrawal reflex was confirmed before surgery. To minimize the risk of postoperative infection, the skin was disinfected with povidone–iodine (Betadine^®^, Avrio Health L.P) and 70% ethanol. Anesthetic depth was assessed every 5 min by monitoring the paw withdrawal reflex until the end of the procedure. Prior to surgery, mice received an ophthalmic solution to prevent corneal drying. Body temperature was maintained at 37 ± 0.5°C using a thermostatically controlled surgical table (model RTC1, Cibertec) with continuous rectal monitoring. A midline dorsal scalp incision was made to expose the skull, and mice were secured in a stereotaxic frame. Four burr holes were drilled: two over the forelimb somatosensory cortex (anteroposterior+0.5 mm, mediolateral ±2.0 mm) for recording electrodes, and two over the visual cortex (anteroposterior −3.0 mm, mediolateral ±2.0 mm) for ground and reference electrodes. Stainless steel screw electrodes with polyimide insulation (PlasticsOne) were inserted and fixed with cyanoacrylate and dental acrylic. Animals were housed individually for recovery.

For somatosensory evoked potential (SSEP) recordings, animals were anesthetized with an intraperitoneal injection of ketamine (100 mg/kg) and xylazine (10 mg/kg). Anesthesia management is critical, as different anesthetics affect the stability of SSEP responses. In our previous experience, isoflurane strongly suppresses evoked potential amplitudes, whereas ketamine/xylazine provides stable responses for at least 30 min [25]. To reduce variability, recordings were performed during the first 10–15 min after confirming the absence of the pedal reflex. Tail-pinch reflex and electroencephalogram (EEG) amplitude were continuously monitored to ensure consistent responsiveness across animals. For evoked potential recordings, the median nerve of each forelimb was stimulated, and evoked potentials were recorded in both hemispheres (contralateral and ipsilateral) in response to the stimulus. For median nerve stimulation, electrodes were placed on the ventral forelimb (wrist) and the proximal biceps brachii. Supramaximal current pulses (typically 1.5 mA, 0.5 ms duration) were applied at 1 Hz. Signals were amplified (×10³) and band-pass filtered (10–2000 Hz) using a portable EMG/EP system (Micromed) at a 16 kHz sampling rate. Sixty responses were averaged, and each average was repeated three times per stimulated limb.

#### Functionality of SDF-1α–SF films in a PT model

The study included four groups: (G01) non-stroke controls (sham), (G02) stroke without treatment, (G03) stroke treated with empty SF films, and (G04) stroke treated with SF films containing 10 ng of SDF-1α. All animals in the four groups underwent a 2 mm × 2 mm craniectomy in the right hemisphere (center of the craniectomy: anteroposterior −1.0 mm, mediolateral +2.5 mm from bregma). SDF-1α-SF films were prepared and reconstituted, and subsequently implanted on the cortical surface 10–12 h later. Craniectomy, film application, and electrode implantation were performed 24 h after PT stroke.

Electrode implantation followed the same SSEP surgical principles, except that electrodes could not be placed over the craniectomy area occupied by the SF film. Consequently, only three electrodes were implanted, allowing recordings from the left, non-infarcted hemisphere.

#### Behavior assessment

Sensorimotor skills were evaluated using the cylinder and grid tests in healthy controls and post-PT stroke subjects. In the cylinder test, mice were placed in a 15 cm × 10 cm beaker, and forelimb contacts during vertical exploration were recorded to calculate a laterality index: [(right − left)/(right + left + both)]. Animals making fewer than 25 contacts in 5 min were excluded. In the grid test, mice walked freely on a 13 mm  × 13 mm metal grid for 5 min; forelimb slips were recorded as failures, and the percentage of failures per limb was calculated relative to total steps. Animals taking fewer than 200 steps in 5 min were excluded.

#### Infarct area and volume determination

Infarct volume in groups G01–G04 was assessed 15 days post-PT. After transcardial perfusion with cold PBS and 4% paraformaldehyde, brains were post-fixed for 1 week and cryoprotected in 30% sucrose. Coronal sections (30 µm) were cut on a freezing microtome (−20 °C), mounted, and stained with toluidine blue (pH 4.0) following standard protocols, including dehydration through graded alcohols, clearing in xylol, and mounting with Dibutylphthalate Polystyrene Xylene (DPX). Coronal sections were digitized, and ischemic areas were measured using ImageJ. Six anatomically matched sections per brain were analyzed, multiplying the damaged area by slice thickness. Infarct volume was obtained by summing across sections and expressed as a percentage of the contralateral hemisphere to account for edema.

### Statistical analysis

Statistical analyses were conducted using SigmaPlot v11.0 (Systat Software, Germany). Data were first assessed for normality (Shapiro–Wilk test) and homogeneity of variances (Levene’s test). For datasets meeting normality and equal variance assumptions, parametric tests were used: Student’s *t*-test, one-way analysis of variance (ANOVA), or two-way ANOVA. *Post hoc* comparisons were performed using Dunnett’s test for one-way ANOVA and Tukey’s test for two-way ANOVA. For data not meeting these assumptions, nonparametric tests were applied: Wilcoxon Mann–Whitney (*t*-test alternative), Kruskal–Wallis (one-way ANOVA alternative), or Friedman test (two-way repeated-measures ANOVA alternative), with Dunn’s multiple comparison test for *post hoc* analyses. Relationships among the variables were assessed using the Pearson product–moment correlation coefficient. The statistical analyses performed are detailed in the figure legends. Statistical significance was set at *P* < 0.05.

## Results

**Figure 1:**
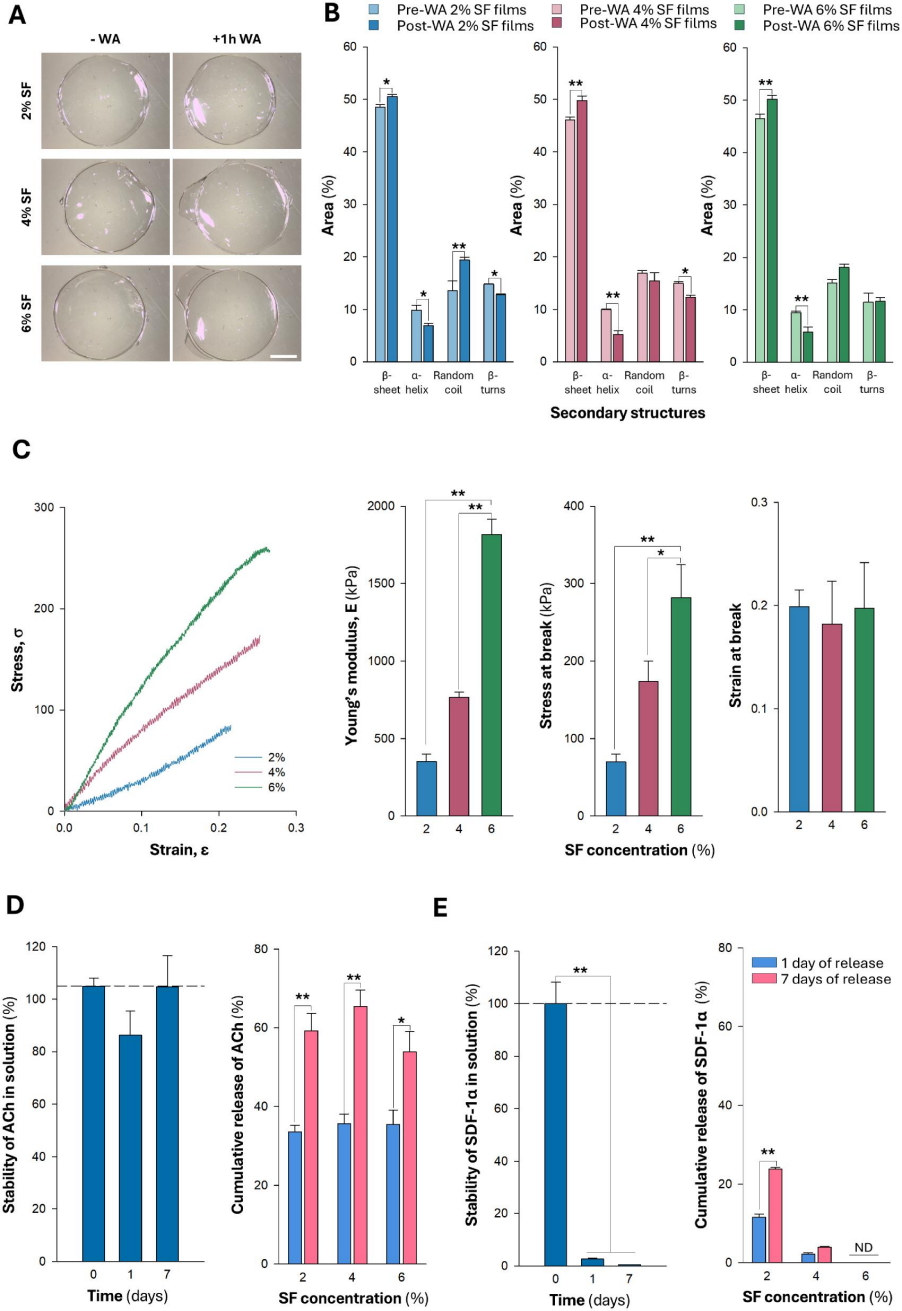
Mechanical, microstructural, and release properties of SF films. (**A**) Representative images of 2%, 4%, and 6% SF films before (–WA) and after (+WA) 1 h water annealing. Scale bar, 1 mm. (**B**) Contribution of various secondary structures to the total area under the Amide I peak, before and after WA. A minimum of 10 films per concentration were analyzed. (**C**) Representative stress (σ)–strain (ε) curves for 2%, 4%, and 6% SF films, along with corresponding Young’s modulus, maximum stress, and maximum strain (left to right, respectively) (*n* = 5). (**D**) Left, stability of ACh measured immediately after mixing with PBS (time 0) and after 1 and 7 days of incubation in PBS (pH = 7.4, 37°C). Right, cumulative ACh released from 2%, 4%, and 6% SF films at 1 and 7 days after molecule encapsulation in the biomaterial (*n* = 8). (**E**) Same experimental design, plotting, and statistical analyses as in (**D**), but for SDF-1α (*n* = 8). Data are presented as mean ± standard error of the mean (SEM). Asterisks indicate statistically significant differences between pre- and post-WA states, between film concentrations, or between different time points. Significance levels were determined by two-way ANOVA for secondary structure distribution and cumulative release, and one-way ANOVA for Young’s modulus, stress at break, strain at break, and molecules stability (*P < 0.05; **P < 0.01).

To develop an implantable material with minimal adverse effects on brain tissue, we reconstituted SF solutions into insoluble films via water annealing (Figure 1A).

Water annealing induced a pronounced structural reorganization in all tested SF concentrations, increasing β-sheets while reducing α-helices (Figure 1B). With the exception of SF2%, random coil structures remained largely stable, while a slight decrease in β-turns was observed in the 2% and 4% SF films. As a result, the films exhibited a high β-sheet content, reaching approximately 50% following water annealing (Figure 1B). Notably, variations in SF concentration did not significantly alter the secondary structures distribution, as similar trends were observed across all conditions. In contrast, mechanical properties were markedly influenced by SF concentration, with films at 6% displaying increased Young’s modulus (∼2000 kPa), tensile strength (∼300 kPa), and strain at break (∼0.20), compared to lower concentrations (Figure 1C). SF films at all tested concentrations remained stable in PBS at 37°C, showing no weight loss or significant changes in β-sheet content before or after water annealing over 1 and 7 days of incubation (Supplementary Figure S1).

Few biological molecules have been identified as potent chemoattractants for MSCs, a stem cell population with considerable therapeutic potential in regenerative medicine. Among these, protein factors such as SDF-1α and MCP-1 have been shown to induce MSCs’ chemotaxis across various tissues. Other stem cell lineages, including HSCs and neural stem cells (NSCs), also respond to SDF-1α-mediated chemotactic signals [26–28]. In addition to protein factors, the neurotransmitter ACh, a non-protein signaling molecule with pleiotropic roles in both the central and the peripheral nervous systems, has also been shown to stimulate MSCs migration [29].

In our experiments, in agreement with previous observations [30], ACh remained relatively stable in PBS at 37°C for at least 7 days, as no statistically significant differences were found across the different time points analyzed (Figure 1D, left), in stark contrast to the rapid and significant decline in SDF-1α concentration observed under the same conditions, which occurred in <24 h (Figure 1E, left). We next evaluated the ability of SF films to sustain the delivery of both molecules. After 7 days, detectable amounts of both molecules were still released from the SF films (Figure 1D, right; Figure 1E, right). Notably, increasing the SF concentration progressively limited the release of SDF-1α, with films prepared at 2% SF achieving the most efficient and sustained delivery (Figure 1E, right). Surface morphology, assessed by scanning electron microscopy, appeared comparable across all film concentrations, with no discernible topographical features that could account for the greater SDF-1α release from 2% SF films (Supplementary Figure S2).

**Figure 2:**
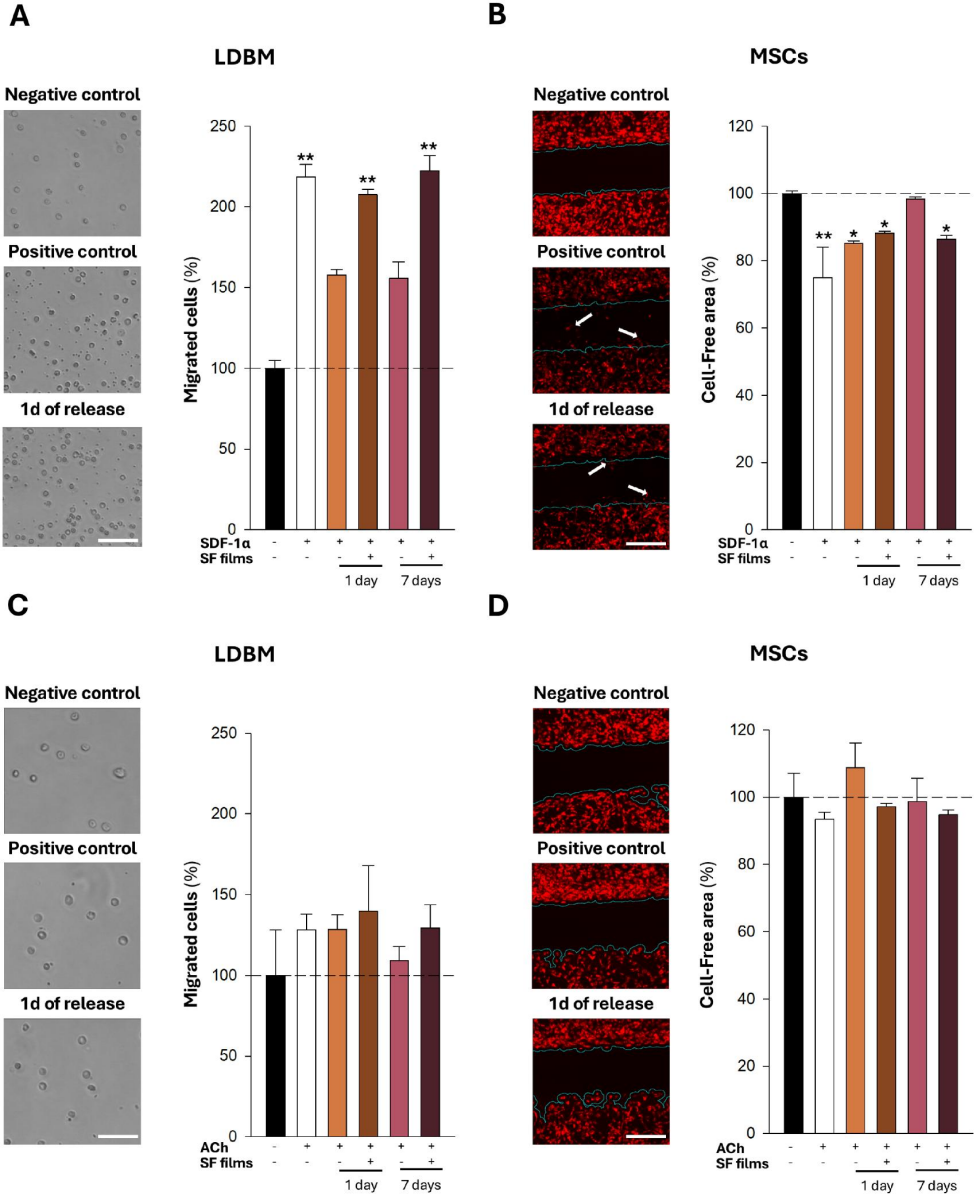
Study of SDF-1-α and ACh functionality *in vitro*. Migration of poorly adherent LDBM (transwell assay) or highly adherent MSCs (scratch-wound test) in response to SDF-1α or ACh. (**A**) Left, representative images of LDBM cells in the low chamber of the transwell under three conditions: (i) no SDF-1α (negative control), (ii) 10 ng freshly added SDF-1α (positive control), and (iii) medium conditioned by SDF-1α-loaded SF films collected after 1 day of release (“1 d release”); scale bar, 500 µm. Right, quantification of the percentage of migrated cells normalized to negative control (dashed line = 100%). The remaining bars represent the positive control (white bar), SDF-1α preincubated in PBS at 37°C for 1 or 7 days, and the release media collected from SDF-1α–SF films after 1 and 7 days of incubation. (**B**) Left, representative wound regions; white arrows highlight cells advancing into the gap; scale bar, 500 µm (left panel). Right, cell-free area (%) normalized to the negative control (dashed line = 100%); lower values indicate greater migratory activity. Panels (**C**) and (**D**) show representative images and quantifications of cell migration, as in (**A**) and (**B**), respectively, but in response to ACh instead of SDF-1α. Data are presented as mean ± SEM (*n* = 8 wells per condition). Asterisks indicate pairwise differences versus the corresponding negative control (*P < 0.05; **P < 0.01; one-way ANOVA).

We assessed the chemotactic potential of the factors SDF-1α and ACh, delivered from 2% SF films, to induce functional responses in MSCs and LDBM (enriched in mononuclear hematopoietic cells, including HSCs and hematopoietic progenitors), using cell-type-specific migration assays (Figure 2). Given the limited adhesive capacity of LDBM cells, transwell assays were used to evaluate their migration (Figure 2A and C), while MSC migration was analyzed using scratch assays (Figure 2B and D).

As shown in Figure 2A and B, freshly prepared SDF-1α alone effectively promoted the migration of both LDBM cells (a higher number of cells in the bottom part of the transwell) and MSCs (as observed by the reduction of the free area), respectively. In contrast, SDF-1α alone preincubated in PBS at 37°C for 1 or 7 days exhibited only moderate chemotactic activity, particularly in MSCs, underscoring the limited stability of this molecule in aqueous solution. Notably, SDF-1α released from SF films, either 1 or 7 days after reconstitution, retained the capacity to induce migration in both cell types, suggesting that SF encapsulation effectively preserves the bioactivity of the protein over time. Conversely, no significant chemotactic response was observed following ACh exposure (Figure 2C and D). Neither freshly prepared ACh nor ACh released from SF films induced migration in either LDBM or MSCs, suggesting, contrary to expectations, that this molecule lacks chemotactic capacity under the tested conditions.

**Figure 3:**
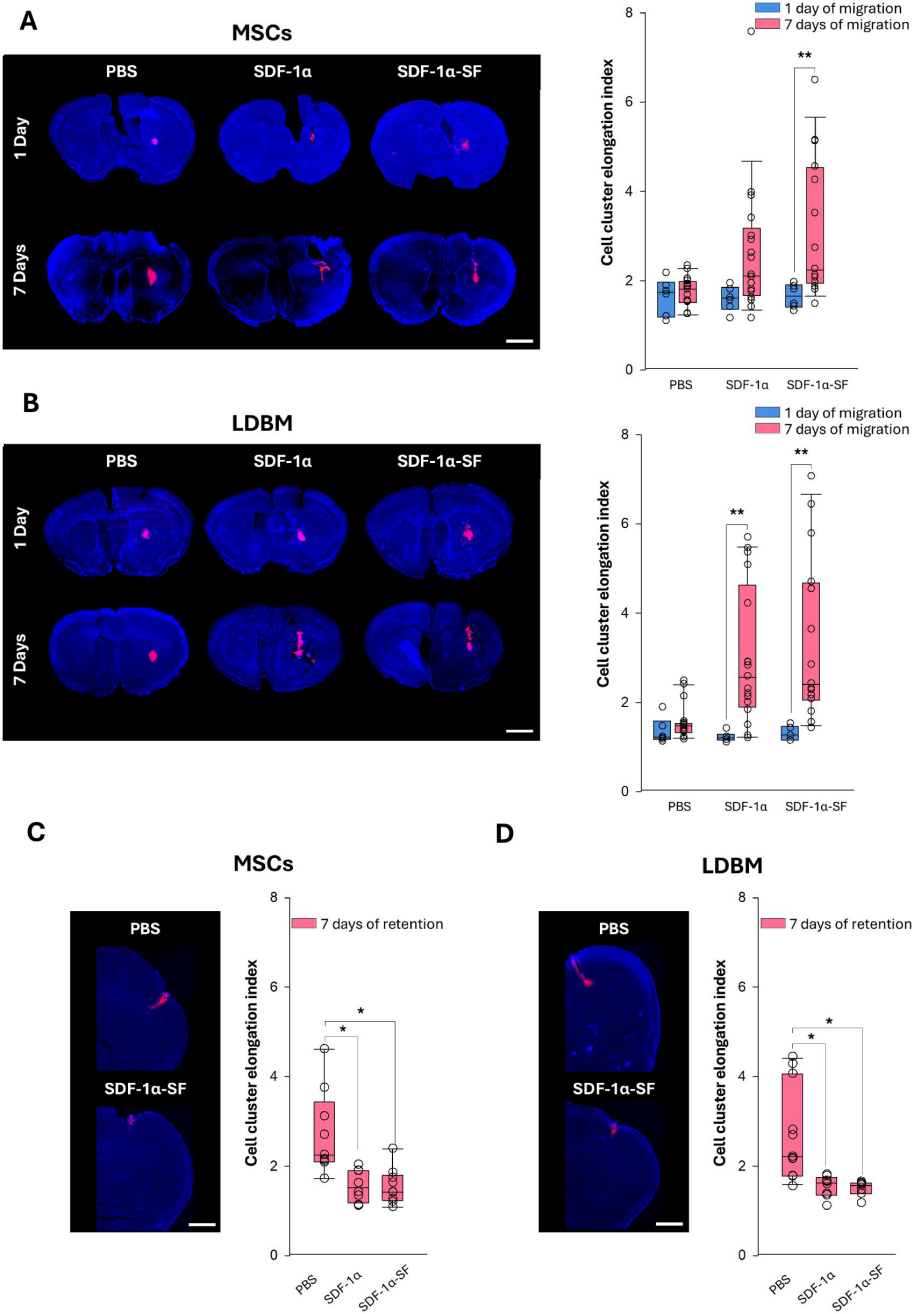
*In vivo* assessment of SDF-1α functionality (migration and retention studies). (**A**) Left, representative DAPI-stained coronal brain sections showing the distribution of DiI-labeled MSCs following striatal injection (3-mm depth from the cortex) and treatment with: (i) PBS (injected at 1-mm depth from the cortex); (ii) fresh SDF-1α (injected at 1-mm depth from the cortex); or (iii) SDF-1α-loaded 2% SF films deposited in the cortical surface. Graft distribution (red area) is shown at 1 and 7 days post-injection (scale bar, 2 mm). Right, quantification of the cell-cluster elongation index at 1 and 7 days post-implantation. (**B**) Same analysis as in (**A**) for DiI-labeled LDBM cells (scale bar, 2 mm). (**C**) and (**D**) Left, representative DAPI-stained coronal sections at 7 days showing retention of DiI-labeled MSCs and LDBM. Unlike the migration assays (**A** and **B**), cells and treatments (PBS, fresh SDF-1α, or SDF-1α-loaded 2% SF films) were delivered on the cortical surface (scale bar, 1 mm). Right, quantification of cell-cluster elongation index at 7 days injection of MSCs and LDBM. All data are shown as mean ± SEM (coronal sections from *n* = 6 mice per group and time point). For migration (**A** and **B**), comparisons used two-way ANOVA. For retention (**C** and **D**), groups were compared using the Kruskal–Wallis test. Statistical significance is indicated by asterisks (*P < 0.05; **P < 0.01).

Following confirmation that SDF-1α–SF films promote chemotactic activity *in vitro*, we next assessed their functional impact *in vivo* after brain implantation. Specifically, we examined two complementary aspects: (i) the ability of DiI-labeled MSCs and LDBM cells to migrate from the striatum (injection site) toward the cortex in response to the chemotactic gradient established by SDF-1α–SF films implanted in the cortex (migration studies) and (ii) the capacity of these films to retain these cell populations within the cortex, thereby limiting their dispersion beyond the targeted region (retention studies). For both experimental paradigms, one group of mice received SDF-1α–SF films implanted on the cortical surface (above the somatosensory cortex), while a control group was administered fresh SDF-1α via direct injection into the same cortical area (Figure 3).

In the migration studies, histological analysis of brain sections obtained 24 h post-implantation of SDF-1α–SF films revealed no significant migration of either MSCs (Figure 3A) or LDBM (Figure 3B), with both cell populations largely confined to the striatal injection site and forming compact, circular clusters of red (Dil fluorescence) signals. However, at one-week post-implantation, the EI, a quantitative parameter reflecting cell/graft displacement [31], was significantly increased in both populations. This morphological shift was accompanied by a transition from a rounded to an elongated spatial distribution, consistent with active, directional migration. This migratory response was particularly enhanced in MSCs from mice implanted with SDF-1α–SF films, compared to those that received soluble SDF-1α alone.

In parallel retention studies, EI values remained consistently low at 7 days following injection of MSCs or LDBM into the somatosensory cortex, directly beneath the site of SDF-1α–SF film or soluble SDF-1α administration (Figure 3C and D). In contrast, animals injected with PBS (no chemoattractant) exhibited significantly higher EI values, indicative of greater dispersion and reduced cell retention. These findings suggest that SDF-1α–SF films not only promote directed migration when delivered at a distance but also support local retention of transplanted stem cells when applied directly at the injection site, maintaining tightly confined cell localization within the target cortical region for at least 1 week following implantation.

As a next step, we investigated the potential of SDF-1α–SF films to recruit circulating endogenous MSCs and HSCs into the brain. However, immunophenotypic analyses (flow cytometry) and functional assays, including the detection of hematopoietic CFU-C and assessment of plastic adherence properties of MSCs, did not allow reliable identification of these cell populations within brain tissue (Supplementary Figures S3–S6). Our outcome was consistent with previous reports and reflects ongoing debate in the field, as the detection of circulating MSCs or HSCs in non-lymphoid organs such as the brain remains technically challenging and controversial [7, 32, 33].

To explore the potential of SDF-1α–SF films to enhance brain recovery after injury, perhaps augmenting the mobilization of endogenous stem cells into the brain, we next assessed the therapeutic efficacy of SDF-1α–SF films in a preclinical model of stroke. A PT model was employed to induce a focal cortical injury, generating a well-defined infarct in the somatosensory cortex of the right hemisphere (Supplementary Figure S7). In this brain injury model, cortical function can be monitored via SSEP recordings, providing a quantitative readout of treatment efficacy.

Before testing the efficacy of SDF-1α–SF films, we first characterized the electrophysiological responses of healthy, non-stroke animals. Stimulation of either the left (Supplementary Figure S8A and B) or the right (Supplementary Figure S8D and E) forepaw reliably elicited robust SSEP in the contralateral hemisphere. These contralateral responses were accompanied by smaller, delayed potentials in the corresponding ipsilateral hemisphere. The amplitude of ipsilateral SSEP showed a significant positive correlation with that of the contralateral responses (Supplementary Figure S8C and F), indicating that unilateral forepaw stimulation primarily activates the contralateral somatosensory cortex, followed by transcallosal propagation to the ipsilateral hemisphere. This secondary response typically emerged with a latency of 5–10 ms, consistent with previous reports [25]. This phenomenon was particularly relevant to our experimental design, as it enabled inference of contralateral cortical activity through analysis of the delayed ipsilateral response.

**Figure 4:**
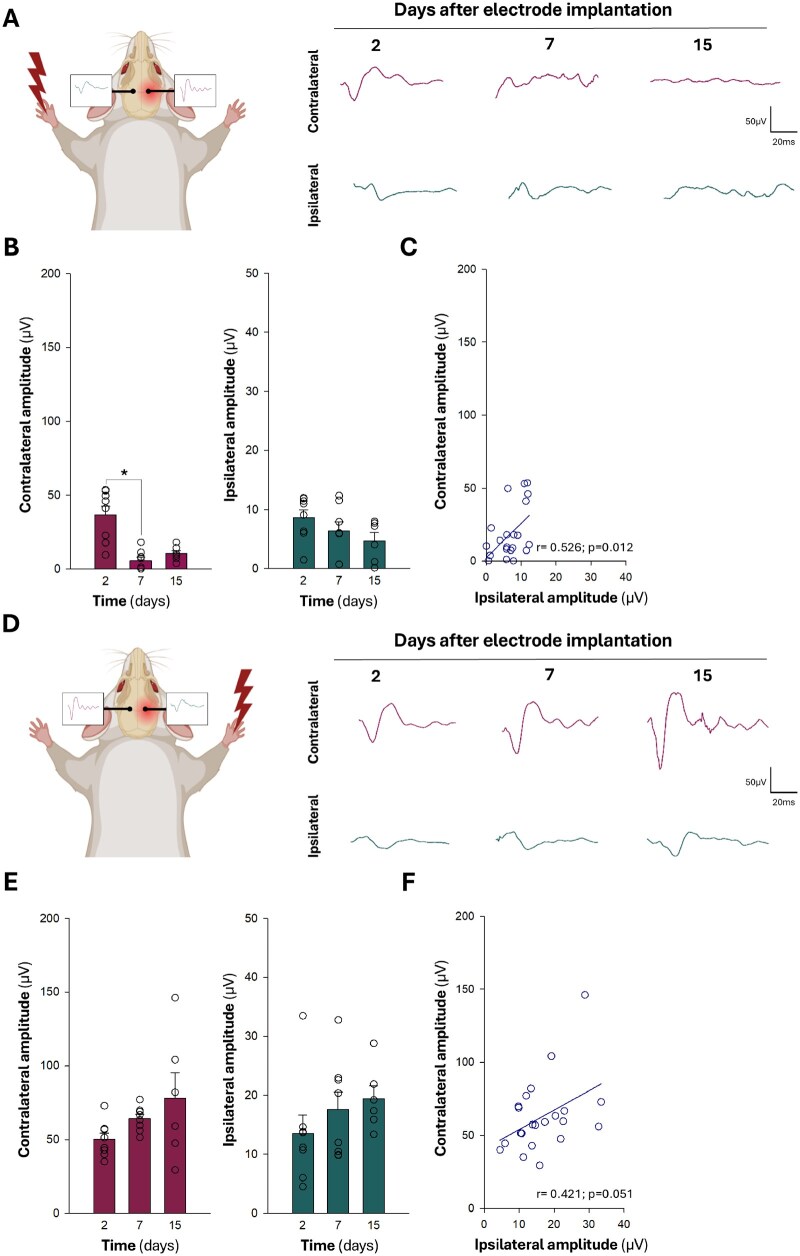
Characterization of somatosensory evoked potentials (SSEP) in a murine stroke model of photothrombosis (PT). (**A**) Schematic illustrating the relationship between left forepaw stimulation and electrode placement in both the contralateral (infarcted) and ipsilateral (non-infarcted) hemispheres. Representative SSEP recorded at 2, 7, and 15 days post-surgery, following electrode implantation 24 h after PT surgery. Note the reduced SSEP amplitudes due to extensive somatosensory cortex damage in the PT model, compared to healthy animals (see Supplementary Figure S8). (**B**) Contralateral and ipsilateral SSEP amplitudes in response to left forepaw stimulation over time after PT. (**C**) Pearson correlation of contralateral and ipsilateral amplitudes over time across all animals. (**D**) Schematic illustrating the relationship between right forepaw stimulation and electrode placement in both the infarcted and non-infarcted hemispheres. (**E**) Contralateral and ipsilateral SSEP amplitudes in response to right forepaw stimulation. (**F**) Pearson correlation of contralateral and ipsilateral amplitudes. Data are presented as mean ± SEM (*n* = 6–8 per group) (*P < 0.05; **P < 0.01; one-way ANOVA).

In contrast, in the PT stroke model, electrical stimulation of the left forepaw resulted in a significant and progressive reduction in evoked potentials within the infarcted right hemisphere across all evaluated time points (2, 7, and 15 days post-surgery) as shown in the representative traces (Figure 4A and B). This decrease was significant as early as day 2 and continued to decline by day 15. Ipsilateral responses recorded in the non-infarcted left hemisphere were also consistently diminished, although with lower amplitudes overall. A positive correlation between contralateral and ipsilateral amplitudes persisted despite these reductions, indicating a preserved but weakened bilateral response pattern (Figure 4C, *r* = 0.526, *P* = 0.012). To assess whether this hemispheric disruption was stimulus-specific, we evaluated evoked potentials following stimulation of the right forepaw, targeting the non-infarcted hemisphere. In this case, contralateral responses recorded in the left (non-injured) hemisphere remained robust across time points, although a mild increase was observed by day 15 (Figure 4D and E). Ipsilateral responses remained consistently low. Notably, the correlation between ipsilateral and contralateral amplitudes under right forepaw stimulation approached statistical significance (Figure 4F, *r* = 0.421, *P* = 0.051), suggesting a potentially coordinated, albeit weaker, response from the non-infarcted hemisphere. These results, consistent with those shown in Supplementary Figure S8, further indicate that ipsilateral activity recorded in the non-infarcted hemisphere reflects functional activity in the somatosensory cortex of the infarcted hemisphere.

**Figure 5:**
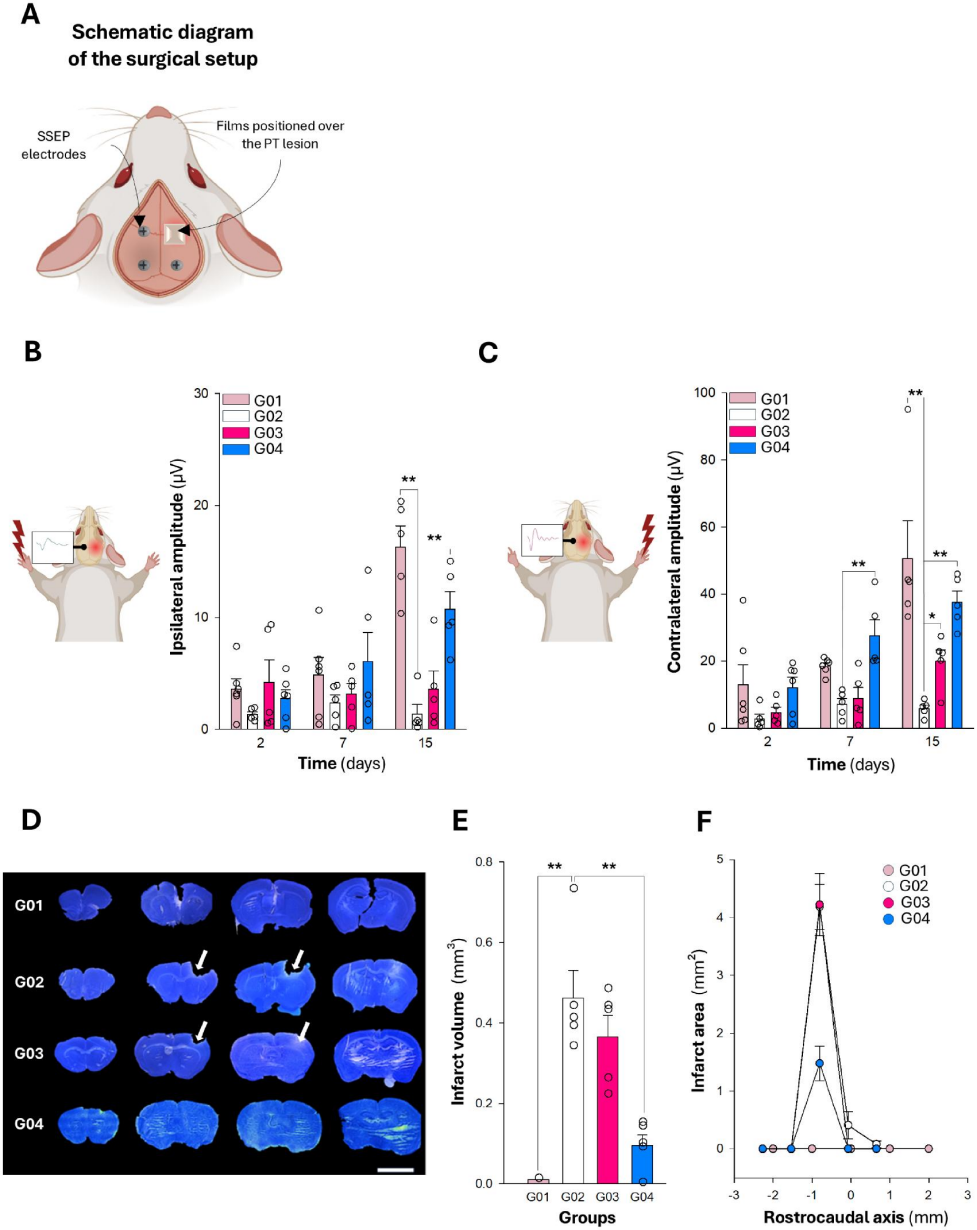
Functional and histological evaluation following SDF-1α–SF treatment in a PT stroke model. (**A**) Surgical schematic: an SF film was positioned over the PT lesion, and electrodes were implanted for SSEP recordings. (**B**) Ipsilateral SSEP amplitude (left-hemisphere recording during left forepaw stimulation) at 2, 7, and 15 days for G01 (sham), G02 (PT untreated), G03 (PT+SF), and G04 (PT+SDF-1α-SF). G04 exhibits higher amplitudes by day 15 compared with G02; G03 shows limited change relative to G02. (**C**) Contralateral SSEP amplitude (left-hemisphere recording during right forepaw stimulation) for the same groups and time points, with increased responses in G04 at 7–15 days versus G02. (**D**) Representative Nissl-stained coronal sections at day 15 showing cortical tissue loss; lesions are smaller in G04 than in G02 and G03. (**E**) Infarct volume (mm³) at day 15 demonstrating reduction in G04 relative to G02. (**F**) Rostrocaudal lesion profile (infarct area, mm^2^) across coronal levels. Data are presented as mean ± SEM (*n* = 5–6 for **B** and **C**; and *n* = 5 for **E** and **F**). Statistical significance versus the untreated PT group (G02). Two-way ANOVA for **B** and **C**; one-way ANOVA for **E** (*P < 0.05, **P < 0.01).

In a separate study, SDF-1α–SF films were implanted directly over the cortical infarcted surface of PT animals (Figure 5A). Importantly, the presence of the film directly over the infarcted cortex precluded direct electrophysiological recordings from the injured hemisphere, as the implant covered the cortical surface. As shown in Figure 5B, the analysis of ipsilateral responses to left forelimb stimulation, which, as mentioned above, reflect the level of residual activity in the opposite, infarcted hemisphere, revealed a progressive increase in amplitude over time in the SDF-1α–SF group (G04, blue bars). By day 15, the ipsilateral responses closely matched those of healthy animals (G01, light pink bars), while PT mice receiving no treatment (G02, white bars) or SF films alone (G03, pink bars) exhibited minimal responses, indicating poor functional recovery. Following right forelimb stimulation (Figure 5C), contralateral evoked responses recorded in the non-infarcted left hemisphere were significantly higher in both healthy animals (G01) and PT mice treated with SDF-1α–SF films (G04) compared to untreated (G02). Significant contralateral evoked responses were also observed at day 15 in mice treated with SF films (G03), while the most pronounced effects occurred in the SDF-1α–SF group (G04), which showed significant improvements at both 7 and 15 days post-treatment, reaching near-control levels.

The recovery of function in the infarcted hemisphere of SDF-1α–SF-treated animals was supported by histological analysis performed after the electrophysiological experiments (Figure 5D). Nissl-stained coronal brain sections showed large infarcts in untreated (G02, white) and SF-only-treated (G03, pink) PT mice, while SDF-1α–SF-treated mice (G04, blue) exhibited substantially smaller infarct areas. Quantitative analysis confirmed a significant reduction in infarct volume in the G04 group compared to both G02 and G03 (Figure 5E). Furthermore, infarct mapping along the rostrocaudal axis revealed a lower peak infarct area in the SDF-1α–SF group, indicating reduced lesion spread and severity (Figure 5F). Despite clear neuroprotective effects at both histological and electrophysiological levels, behavioral improvements were generally not observed within the post-stroke period. Sensorimotor performance, assessed by the cylinder and grid-walking tests, remained largely unchanged across time points and PT groups. However, a significant improvement was observed at day 7 post-treatment in both SF and SDF-1α–SF groups, as indicated by a reduction in slips of the left forepaw contralateral to the infarcted hemisphere (Supplementary Figure S9).

## Discussion

Treating acute brain injuries such as stroke or trauma remains a major clinical challenge. A key limitation lies in the incomplete understanding of secondary injury mechanisms in humans compared to animal models, which may partly explain the failure of many neuroprotective agents in clinical trials despite robust preclinical efficacy. Additionally, several compounds with promising effects in animal models, such as NXY-059 suffer from poor blood–brain barrier permeability, preventing them from reaching therapeutic concentrations in human brain tissue. For instance, NXY-059 reduced infarct size and improved outcomes in preclinical stroke models but failed in Phase III trials, possibly due to its limited brain penetration [34, 35]. In another example, the molecule gavestinel (GV150526), an N-methyl-D-aspartate (NMDA) receptor antagonist, showed efficacy in animal models but, like NXY-059, failed to translate successfully in humans [36, 37]. Moreover, some experimental protocols in animals rely on delivery methods or cell therapies that are difficult to replicate clinically. Therefore, achieving meaningful preclinical-to-clinical translation requires therapies that are not only effective but also minimally invasive and compatible with routine human use.

The chemokine SDF-1α is a potent chemoattractant involved in cell migration and positioning across various tissues. For example, it plays key roles in HSCs’ retention within the bone marrow and in guiding neural progenitor migration during nervous system development [38]. While the use of SDF-1α to recruit neural or bone marrow-derived stem cells to sites of brain injury holds therapeutic potential, it may also promote the infiltration of inflammatory cells from the bloodstream into the brain. In this regard, the literature presents conflicting findings on the role of peripheral immune cells in post-stroke damage progression. Some studies suggest a protective role; for instance, SDF-1α derived from the brain endothelium has been shown to attract natural killer cells that contribute to neuroprotection during ischemic stroke [39]. Conversely, blocking neutrophil migration with Reparixin, a CXCL8 receptor inhibitor, has also been associated with reduced brain injury and improved recovery in rodent models [40]. Other reports indicate that suppressing neutrophil infiltration, such as by targeting CXCR2, does not necessarily result in functional improvement post-stroke [41].

Several additional preclinical studies have investigated the therapeutic potential of SDF-1α for the treatment of brain stroke. For example, the administration of fresh SDF-1α alone reduced infarct size in a rat model of brain ischemia/reperfusion [42], an effect we also observed in the PT mouse model following injection of SDF-1α alone (data not shown). In another study, endothelial progenitor cells engineered to overexpress SDF-1α significantly improved functional outcomes after stroke in the middle cerebral artery occlusion (MCAO) model [43]. Similarly, intraventricular delivery of SDF-1α decreased infarct area and enhanced recovery in a permanent MCA occlusion model in rats [44]. Reported mechanisms include recruitment of bone marrow-derived cells, promotion of angiogenesis, and improved cerebral blood flow in the infarcted hemisphere [42]. However, opposing findings also exist, since pharmacological inhibition of SDF-1α signaling improved sensorimotor outcomes without affecting infarct size after transient MCA occlusion [45].

Although these previous studies illustrate the possible therapeutic potential of SDF-1α, its clinical efficacy in stroke remains unproven due to pharmacokinetic and biological challenges. We and others have shown that SDF-1α has a short half-life and is quickly degraded [18, 46]. Thus, systemic administration of SDF-1α might result in widespread biodistribution, reducing its concentration at the ischemic region and increasing the risk of off-target effects. To avoid frequent injections and maintain therapeutic levels of SDF-1α, encapsulation within biomaterials can shield SDF-1α from inactivation under physiological and inflammatory conditions, ensuring its sustained presence at the target site and prolonging delivery during the critical window when remodeling brain changes linked with recovery are required.

To address these conceptual limitations, in this study, we designed an implantable SF film capable of delivering this chemoattractant molecule. SF, a natural biomaterial derived from silkworm silk, has garnered considerable attention for its outstanding properties in clinical applications. Beyond sutures, SF can be processed into various formats: films, hydrogels, scaffolds, nanoparticles, and coatings, enabling a wide range of medical applications. Its versatility, combined with favorable interactions with biological tissues, has driven growing interest in its therapeutic potential. Earlier findings demonstrated that SF can be safely applied in the CNS [16]. These findings have also been corroborated by seminal studies of SF implanted in the rat brain, which consistently reported favorable biocompatibility [47]. Subsequent studies demonstrated the capacity of SF to support cell growth, facilitate tissue remodeling, and serve as a carrier for therapeutic agents, pointing to its significant potential in addressing brain injuries, neurodegenerative diseases, and spinal cord injuries [47–49].

Various alternative biomaterials have been developed to enable the delivery of distinct therapeutic molecules including SDF-1α into the brain. The most commonly used formats for drug administration in injured brain tissue are nanoparticles and hydrogels. For example, intracerebral delivery of SDF-1α encapsulated in pH-sensitive poly(urethane amino sulfamethazine) (PUASM) micelles into the striatum of rats subjected to permanent ischemia promoted both neurogenesis and angiogenesis [50]. Nanohybrid hydrogels composed of sulfated glycosaminoglycan-based polyelectrolyte complex nanoparticles have been used to deliver SDF-1α and basic fibroblast factor, which enhanced neurogenesis, angiogenesis and chemotaxis of NSCs toward the infarct core in an *in vivo* ischemic stroke rat model [51]. In another study, syndecan-4 and SDF-1α functionalized enhanced the binding, spreading and differentiation of endothelial colony-forming cells to endothelial cells [52]. SDF-1α has also been applied from heparin nanoparticles, leading to a revascularization in the stroke core of mice, in addition to neural progenitor cells recruitment and migration to the infarcted area and reduction of injury size [53]. SDF-1α covalently grafted on the surface of extracellular vesicles promoted NSC recruitment and reduced brain atrophy in rats undergoing transitory MCAO [54].

Hydrogels are of particular interest for implantation in soft tissues such as the brain. Certain types can be injected in liquid form (pre-gel) and gel *in situ*, thus minimizing injection-induced damage. Natural polymers such as gelatin, hyaluronic acid (HA), and collagen have been frequently used. For instance, HA-based hydrogels loaded with Brain-derived Neurotrophic Factor (BDNF), Vascular Endothelial Growth Factor (VEGF), or angiopoietin-1 injected into the infarct cavity at different post-ischemia time points enhanced neurogenesis, angiogenesis, and functional recovery [55–57]. Similarly, alginate [58], collagen [59] or commercial hydrogels [60] releasing BDNF and VEGF reduced infarct volume and exerted anti-inflammatory effects by attenuating astrogliosis and microgliosis. Previous studies have demonstrated the capacity of SF hydrogels to deliver SDF-1α with high [61] and, contradictorily, low [18] efficacy. SDF-1α has also been encapsulated and delivered from Ac-(RADA)\_4_-CONH_2_ self-assembling peptide (RADA) and HA hydrogels [62].

In our experiments, SDF-1α was released more efficiently from SF films than from SF hydrogels [18]. This difference likely reflects the more restricted permeability of SF hydrogels, which limits the diffusion of molecules entrapped within their internal pores, whereas in films, SDF-1α is probably distributed closer to the surface and the release medium. Our study observed a progressive reduction in SDF-1α release with increasing SF concentration, which is consistent with findings in the literature. Higher polymer concentrations produce denser and more compact SF films, which can increase the tortuosity of diffusion pathways and hinder the transport of molecules to the surface. This is conceptually consistent with the model proposed by Hines and Kaplan [63], who demonstrated that the entrapment of large FITC-dextrans within SF films results from limited pore interconnectivity and restricted diffusion paths, whereas smaller dextrans diffuse more freely. In our study, this may partly account for the reduced release of SDF-1α (MW: 8.0 kDa) compared to the smaller molecule ACh (MW: 146.2 Da), whose release remained consistent across all formulations. Notably, these effects occurred despite similar β-sheet content across all SF concentrations, suggesting that the slower release at higher concentrations was primarily driven by structural constraints and matrix density rather than crystallinity. Still, other studies have shown that SF crystallinity can influence molecular release [64].

Importantly, we demonstrated for the first time that epicortical implantation of SDF-1α-loaded SF films significantly reduces infarct size following brain ischemia. Given that freshly reconstituted SDF-1α undergoes rapid degradation, with over 60% of the protein degraded within just 2 h [18], and considering that the SDF-1α-SF films were implanted between 10 and 12 h post-reconstitution, the observed *in vivo* therapeutic effect appears closely linked to the SF matrix’s ability to preserve SDF-1α bioactivity, as supported by our *in vitro* migration assays. Although treatment with SDF-1α–SF films did not produce general improvements in sensorimotor function as measured by behavioral tests, electrophysiological assessments confirmed recovery of somatosensory cortex function. Notably, evoked potential responses in PT mice treated with SDF-1α-SF films were comparable to those observed in non-ischemic control animals. These findings are consistent with the hypothesis that the effect of SDF-1α-SF films was primarily neuroprotective when these therapeutic scaffolds were implanted in the first 24 h after PT.

On the other hand, although the reduction was not statistically significant, we observed a slight decrease in infarct volume in PT mice treated with SF alone. This effect was associated with partial recovery of contralateral SSEP amplitude by day 15, as well as significant improvement in the grid-walking test at day 7 post-treatment. However, ipsilateral activation in the non-infarcted hemisphere, which reflects the activity of the infarcted hemisphere, remained unchanged and comparable to that of untreated PT animals. Notably, cortical recovery assessed by SSEP was more pronounced in both contralateral and ipsilateral responses when SF was combined with SDF-1α. Several studies have reported the intrinsic cytoprotective properties of SF. Specific SF-derived peptides, such as brain factor 7 (BF7), have been shown to reduce reactive oxygen species and thereby promote cytoprotection [65]. Evidence from brain injury and stroke models suggests potential cytoprotective effects of SF and its derivative peptide BF7, but the underlying mechanisms remain unclear [66, 67]. The antioxidant activity of SF has been demonstrated in zebrafish cell models, where it has been hypothesized that the β-sheet structure of SF possesses H_2_O_2_ scavenging properties, undergoing a transition into random coil structures upon oxidation [68]. The demonstrated biocompatibility of this material in the nervous system [16, 47], along with its modulatory effects on inflammation and oxidative stress, likely contributes to the absence of adverse responses following its epicortical implantation. No significant changes in body weight, spontaneous behaviors, or responses to external stimuli were observed during routine daily monitoring, indicating that all experimental groups tolerated the implanted SF films well throughout the 15-day evaluation period. Importantly, no overt epileptic seizures were detected, nor were there signs of serious health problems or welfare loss that could arise from neurological complications or infections associated with the implanted material.

Similar SF-based formats have previously been implanted epicortically for localized delivery of antitumor agents [69]. Other epicortical implants, specifically hyaluronic acid/methylcellulose (HAMC), have also been explored for drug delivery. For instance, delivery of Epidermal Growth Factor (EGF) or BDNF from HAMC hydrogels stimulated neurogenesis, plasticity, and sensorimotor recovery [70, 71]. Surface implantation of biomaterials may seem invasive, but it enables controlled, localized delivery, especially valuable in brain pathologies, and the implant can be easily removed after treatment if needed. Clinical procedures such as decompressive craniectomy (DC), commonly performed in 1–5% of severe ischemic stroke patients and 10–30% of those with TBIs, create an opportunity for surface-accessible interventions. Though DC carries surgical risks, it is often justified when intracranial pressure cannot be otherwise managed. Less invasive alternatives like burr hole drilling also offer access routes to the brain surface, potentially facilitating the delivery of therapeutic biomaterials or cell-loaded constructs in a safe and targeted manner. To date, administration of biomaterials for stroke treatment has not reached clinical application, although biocompatibility of different formulations has been tested in non-human primates [55, 72].

While several other biomaterials also demonstrate good compatibility, the main advantages of SF likely lie in its high stability and limited degradability within neural tissue, even under conditions of pronounced neuroinflammation, such as those observed in stroke or neurodegenerative diseases [17]. Furthermore, SF can be readily sourced and scaled up for industrial production at relatively low cost, as exemplified by its use in surgical sutures, thereby avoiding the drawbacks associated with animal-derived or synthetic materials. For therapeutic contexts requiring shorter time windows, other equally biocompatible materials with faster degradation in the nervous system may be preferable such as collagen [17].

An additional observation from this study is that, despite using different methodologies aimed at examining the functional features of hematopoietic progenitors (i.e. their ability to produce CFU-C) and MSCs (i.e. their strong adhesion capacity), we were unable to detect or distinguish these populations from those of neural origin. Reviewing the literature, no evidence has shown a physiological or pathological migration of endogenous HSCs or MSCs to the brain, as most studies have explored the ability of infused stem cells (marked with different fluorescent tracers) to traffic into the brain. While the identification of specific markers in HSCs and MSCs remains a rapidly evolving field, two of their most relevant functional properties, the colony-forming ability of hematopoietic progenitors and the strong adherence capacity of MSCs, have not been demonstrated in either healthy or ischemic brains following endogenous mobilization. Furthermore, these functional hallmarks have also not been observed in brains previously implanted with MSCs or HSCs. One possible explanation may involve the influence of molecular signaling and interactions with resident brain cells, which could impair or modulate these functions *in vivo*, despite their robust expression *in vitro*. For instance, it has been previously reported that signs of toxicity in MSCs in contact with neural media with long culture times superior to 12 h [73]. Our findings and current literature raise questions about the true ability of these stem cell types to mobilize endogenously into the brain after injury such as stroke. Alternatively, this apparent absence could reflect methodological limitations in detecting and distinguishing cells of different origin within brain tissue.

Further limitations of this study should be considered. First, the mechanisms underlying the neuroprotective effects of SDF-1α-SF films remain unknown. Previous studies have shown that SDF-1α exerts pleiotropic actions after stroke, promoting neuroprotection, neurogenesis, and angiogenesis, as well as recruiting progenitor cells and activating repair pathways [74, 75]. An intriguing possibility is that this molecule may also promote collateral branching, thereby improving blood flow and perfusion in peri-lesional tissue, an underexplored effect in stroke, although SDF-1α has been shown to stimulate arteriogenesis in the heart [76]. Second, technical constraints limit the possibility of long-term evaluation. PT was induced first, followed 24 h later by implantation of the SF films and recording electrodes, after which the cranial window was sealed with dental cement. The complexity of these procedures, combined with longitudinal monitoring using functional tests and electrophysiology, made it difficult to sustain recordings beyond two weeks, as stability typically declined after the third week in our hands. Despite the 15-day follow-up, the use of an objective electrophysiological method, rarely applied in small animals, demonstrated that SF combined with SDF-1α promotes functional recovery. Third, although other stroke models, such as the MCAO model, are clinically relevant and recommended by STAIR guidelines [77], the PT model offers several advantages: (i) high reproducibility in lesion size and location, and (ii) precise targeting of cortical areas. The PT model allowed direct placement of the SF films on the lesioned cortex, whereas lesion extent and location are more variable in the MCAO. Compared with the MCAO model, the PT model differs in the nature and extent of vascular occlusion, and there is ongoing debate about the presence of an ischemic penumbra: some authors report almost no penumbra due to rapid necrosis [78], while others observe a reduced but detectable penumbra [79]. The larger ischemic penumbra produced by the MCAO model could offer an opportunity to study the potential neuroprotective effects of SDF-1α-SF films. However, due to the greater variability in infarct size and location in the MCAO model, brain imaging–based pre-evaluation would be required to adjust both the dimensions and placement of the films to the specific lesioned area. Importantly, we previously found that both stroke models (MCAO and PT) reproduce nonconvulsive seizures during the hyperacute stage [80], paralleling clinical signs seen in a small but significant proportion of stroke patients, which supports the translational relevance of the PT model. Fourth, complementary behavioral tests are needed to better interpret the overall lack of recovery at the behavioral level. We previously reported that the grid-walking and cylinder tests are particularly sensitive for detecting coordination deficits in MCAO animals with cortical damage [25]. Notably, despite the generally limited behavioral improvements observed in our study with the PT model, a significant effect was detected in the grid-walking test at day 7 post-treatment, suggesting that subtle or transient functional recoveries may occur that are not consistently captured across all sensorimotor assays. Controversial issues have been raised regarding the relationship between infarct size and functional recovery in rodents, as a reduction in infarct volume does not necessarily translate into behavioral improvement. Indeed, certain behavioral tests may yield positive outcomes that are not consistently replicated across alternative assessments, and in many cases, the recovery timeline varies substantially depending on the test employed [57]. For example, it has been reported that SSEP and BOLD measures of functional brain activation correlate poorly with behavioral outcomes after ischemia in rats [81]. While electrophysiological measures such as SSEP provide a robust and quantitative assessment of cortical function after stroke and treatment, we currently do not have a definitive explanation for why the reduced infarct size observed in mice treated with SDF-1α–SF films did not translate into a consistent pattern of functional recovery. The 2-week behavioral assessment window may have been too short to reveal functional improvements, even in the presence of a smaller infarct. In a previous study by our group, MCAO animals treated with stem cells encapsulated in SF hydrogels exhibited increased evoked potential activity as early as the first week post-treatment, but behavioral improvements in the cylinder and grid-walking tests only appeared after five weeks [48]. Future studies will be required to assess long-term behavioral recovery, either using the same behavioral tests applied in the present work or complementary assays such as the pasta handling test.

In summary, this study, in line with strategies involving the release of trophic factors from stem cells embedded in biomaterials, advances the design of biomaterial platforms aimed at better controlling encapsulated molecules. The evidence gathered in this study supports the ability of SF films to extend the lifetime of SDF-1α. Although the present study illustrates the capacity of this factor encapsulated in SF films to reduce the injury area during the neuroprotective window (acute post-stroke stage), further research is needed to explore the potential of this biomaterial-based system to preserve the structural integrity and biological function of this labile molecule during extended treatment windows. For example, additional research should focus on the capacity of SF films to support continued recruitment and therapeutic function of stem cells to stimulate the rewiring of peri-lesional areas, enabling these regions to assume control of impaired functions while maintaining their native roles. Indeed, peri-lesional areas exhibit increased neuroplastic potential within the first 4–8 weeks post-stroke, facilitating partial functional reassignment. Early rehabilitation interventions during this window are thought to enhance peri-lesional remapping, thereby supporting improved functional outcomes [82]. While the primary objective of our work was to provide proof-of-concept evidence demonstrating the feasibility of using implanted SF films to deliver neuroprotective molecules directly onto the injured cortical surface, future mechanistic studies and longer-term evaluations will be necessary to clarify their specific effects in stroke and to support regulatory preclinical assessments prior to clinical translation. Given the broad range of modulatory effects attributed to SDF-1α [42–45], subsequent investigations should prioritize three key aspects. First, the analysis of early inflammatory responses, assessing astrocytic (GFAP+) and microglial (Iba-1+) reactivity and inflammatory mediators including Inducible Nitric Oxide Synthase (iNOS)-derived Nitric Oxide (NO), Tumor Necrosis Factor Alpha (TNF-α), Interleukin-1βeta (IL-1β), and Interleukin-6 (IL-6), which SDF-1α has been shown to modulate in TBI models [75]. In parallel, oxidative stress could be evaluated using fluorescent probes for reactive oxygen species (e.g. dihydroethidium, DHE) [83]. Second, the exploration of neurogenesis cues, which are essential for promoting peri-lesional plasticity, examines at later time points the proliferation and recruitment of neural progenitors (DCX+/BrdU+) from the Subventricular Zone (SVZ) and Hippocampal Subgranular Zone (SGZ) toward peri-lesional areas and their differentiation into new neurons (NeuN+/BrdU+) [42, 43, 57, 84]. Finally, the examination of the angiogenic capacity of SDF-1α–SF films to promote vascular remodeling after stroke [42, 43], through analysis of peri-infarct vasculature and angiogenic markers (CD31+/BrdU+), complemented by functional assessments of cerebral blood flow and reactive hyperemia.

## Conclusions

This study highlights the potential of SF films as an effective platform for the controlled release of SDF-1α, an unstable chemokine of considerable interest for tissue engineering in both cerebral and non-cerebral contexts. The significance of SDF-1α lies in its well-documented properties: promoting chemotaxis, recruiting a broad range of stem and differentiated cells, and remodeling existing vasculature to enhance collateral blood flow. By preserving the bioactivity of SDF-1α, our SF-based scaffold maintained its chemotactic function for at least seven days post-reconstitution, promoting the migration of hematopoietic mononuclear cells (including HSCs) and MSCs both *in vitro* and *in vivo*.

While the precise mechanisms underlying the observed neuroprotective effects, namely, infarct size reduction and preservation of somatosensory cortical activity, remain to be elucidated, the data support the rationale of combining biomaterials with SDF-1α for CNS neuroprotection. Notably, our approach may enable the endogenous mobilization of substantial numbers of mesenchymal and HSCs from reservoirs such as bone marrow or adipose tissue, potentially facilitating brain recruitment without the need for cell transplantation or *ex vivo* manipulation. However, whether such mobilization into the brain truly occurs remains an open question, possibly limited by biological constraints or detection sensitivity.

Additional work is needed to evaluate the long-term stability of SDF-1α within the SF matrix and to determine whether this strategy can support sustained recruitment and integration of reparative cells over clinically relevant timeframes (i.e. once damage is established, during the subacute stroke stage). Nonetheless, this approach reinforces the promise of combining biomaterial scaffolds with bioactive molecules to overcome pharmacokinetic barriers and stimulate endogenous repair pathways, providing a foundation for future regenerative strategies for CNS injuries.

## Supplementary Material

rbaf129_Supplementary_Data
