# Supplementary material for: Stabilization of CXCL12 (SDF-1α) via silk fibroin films enhances stem cell migration/retention and functional recovery after stroke
Source: Regen Biomater. 2025 Dec 12;13:rbaf129. doi: 10.1093/rb/rbaf129 (PMC12860479; doi:10.1093/rb/rbaf129)
Supplement: rbaf129_Supplementary_Data [file rbaf129_supplementary_data.docx]

**ºSUPPLEMENTARY MATERIALS AND METHODS**

**Stabilization of CXCL12 (SDF-1α) via Silk Fibroin Films Enhances Stem Cell Migration/Retention and Functional Recovery After Stroke**

Amira Lekouaghet, Marta Sánchez-Díez, José Pérez-Rigueiro, Francisco J. Rojo, Carmen Ramírez-Castillejo, Yolanda Ruiz-León, Fivos Panetsos, Gustavo V. Guinea and Daniel González-Nieto

Supplementary information consists of:

1. Supplementary Methods
2. Supplementary Figure S1. Stability of different SF films concentrations over time in PBS at 37 ºC
3. Supplementary Figure S2. Scanning electron microscopy images of silk fibroin films at increasing concentrations
4. Supplementary Figure S3. Reduced adhesion and proliferation of MSCs in contact with brain-derived cells
5. Supplementary Figure S4. Reduced adhesion of MSCs after transient *in vivo* exposure to brain tissue
6. Supplementary Figure S5. Flow-cytometric detection of MSCs among brain-derived cells
7. Supplementary Figure S6. Colony-forming unit (CFU) assay to detect hematopoietic progenitor cells in bone marrow, blood, and brain-derived preparations
8. Supplementary Figure S7. Characterization of the photothrombotic (PT) stroke model targeting primary somatosensory cortex (S1)
9. Supplementary Figure S8. Correlation between ipsilateral and contralateral somatosensory evoked potentials (SSEP) in healthy mice
10. Supplementary Figure S9. Behavioral assessment of motor asymmetry following SDF 1α–SF film treatment in a photothrombotic (PT) stroke model

**Supplementary methods**

1. *In vitro evaluation of SDF-1α and Acetylcholine-induced migration in poorly adherent hematopoietic mononuclear cells*

The biological functionality of SDF-1α and ACh released from SF films was evaluated through the migration assay of low-density bone marrow (LDBM) cells using a transwell system. LDBM cells (which contain lymphocytes, monocytes and HSCs and progenitors) were isolated from CD1 mice (Charles River Laboratories) aged 2 to 3 months. Under sterile conditions, the pelvic and lower limb bones (femurs and tibias) were harvested and crushed in cold PBS. The cell suspension was filtered through a 100 μm cell strainer and centrifuged at 1500 rpm for 5 minutes. The resulting cell pellet was resuspended in 5 mL of DMEM supplemented with 10% fetal bovine serum (FBS) and 1% penicillin-streptomycin (P/S) and layered onto 5 mL of Histopaque-1083 (Sigma-Aldrich, 10831). This suspension was centrifuged at 1700 rpm without brake for 30 minutes at room temperature. The white interface was carefully aspirated and washed with 40 mL of PBS, followed by centrifugation at 1500 rpm for 5 minutes to remove any remaining Histopaque residues. The LDBM cells were then resuspended in assay medium (DMEM supplemented with 0.5% BSA and 1% P/S).

For the migration assay, 1 × 10^5^ LDBM cells in 100 μL of assay medium were added to the upper chamber of Transwell plates (Costar 3422), while the lower chamber contained 600 μL of medium corresponding to the control or treatment condition. Experimental groups included: negative controls (−SDF-1α or −ACh), positive controls consisted of media supplemented with 10 ng of each chemoattractant (SDF-1α or ACh) added to 600 µL of culture medium, and treatment groups exposed to SDF-1α or ACh released from SF films at 2%, 4%, or 6% concentrations, with release times of 1 and 7 days. After 4 hours of incubation, the cells that migrated to the lower chamber were counted to assess the chemotactic response.

2. *In vitro evaluation of SDF-1α and Acetylcholine-induced migration in highly adherent MSCs*

MSCs were isolated and expanded following a previous methodology [1]. MSCs (passages 5–8) were cultured in complete medium (DMEM supplemented with 10% FBS, 1% penicillin-streptomycin, and 2 mM L-glutamine) under standard conditions (37°C, 5% CO₂, humidified). To facilitate imaging, cells were labeled with Vybrant DiI (Invitrogen™, V22885). Briefly, 1 × 10^6^ MSCs were suspended in 1 mL PBS and incubated with 5 µM DiI for 20 minutes at 37°C, followed by two PBS washes to remove unbound dye.

A scratch migration assay was performed using Ibidi inserts (Cat# 80209) to generate a defined 500 µm cell-free gap. Each compartment of the insert was seeded with 1 ×10^4^ DiI-labeled MSCs in 70 µL complete medium. After 24 hours, once cells reached confluence, inserts were removed and fresh medium corresponding to the control or treatment conditions (same groups as in the LDBM migration assay) was added. Images were captured (at 0 and 4 hours after insert removal) using an inverted microscope (Leica, Mannheim, Germany), and the cell-free area was quantified using ImageJ software to assess migration.

3. *Assessment of endogenous stem cells mobilization towards the brain*

To investigate endogenous recruitment of stem cells within the brain, a comprehensive strategy was developed based on three key cellular features: adhesion properties of MSCs, surface marker expression of MSCs and LDBM, and functional colony-forming capacity of LDBM.

According to the International Society for Cellular Therapy [2], MSCs are known for their strong adherence to plastic surfaces, which allows enrichment by selective removal of weakly adherent or non-adherent cells during graded trypsinization. *Ex vivo* MSCs were pre-labeled with DiI and either cultured alone or co-cultured (1 : 1) with non-fluorescent brain-derived cells. Brain cells were obtained by mechanical dissociation of brain tissue in cold PBS, followed by filtration and preparation for culture. After 24 hours, non-adherent cells were removed, and graded trypsinization was applied to detach less adherent cells. Images were acquired at different time points (range 0-60 seconds) to assess MSCs adhesion in mono- and co-culture. In a complementary approach, pre-labeled MSCs with Dil were transplanted into the brain; after 24 hours, brains were processed using the same dissociation and culture protocol to evaluate MSCs detectability (strong adhesion) within brain-derived populations.

Flow cytometry was performed to characterize MSCs and to determine potential phenotypic overlap with brain-derived cells. According to previous immunophenotypic characterization [3], MSCs (~1 × 10^6^ cells) were stained with a panel of four markers: CD45, CD31, Ter119 (to exclude hematopoietic, endothelial, and erythroid lineages), and CD51 (integrin αV) as a positive MSCs marker (antibody concentrations in Table 1). Cells were incubated with the antibody panel for 30 minutes at 4°C, fixed with 4% paraformaldehyde, washed, filtered through a 40 μm mesh, and analyzed on a BD FACSCanto II cytometer; data were processed using FlowJo v10.2.

Brain tissue from CD1 mice was harvested, meninges removed, and tissue minced (<1 mm fragments). Two dissociation approaches were compared: (i) enzymatic digestion with papain (30 min, 37°C) followed by mechanical trituration, and (ii) purely mechanical dissociation by compressing tissue between frosted slides. Suspensions were filtered (100 μm) and centrifuged. Myelin was removed, and mononuclear cells enriched using a 30% Percoll gradient (700 × g, 30 min, no brake), followed by washing and resuspension for flow cytometry. Once the marker panel and dissociation protocol were validated and phenotypic overlap excluded, MSCs were transplanted into the brain. Brain samples were subsequently analyzed by flow cytometry to assess the presence and phenotype of transplanted MSCs.

Supplementary Table S1. Antibodies used for flow cytometry analysis

| Antibody | Manufacturer | Cat. Number / Final dilution | Purpose |
| --- | --- | --- | --- |
| PE Rat Anti-Mouse CD45 | BD Pharmingen™ | 561087 / 1:100 | Hematopoietic lineage exclusion |
| PE Rat Anti-Mouse CD31 | BD Pharmingen™ | 561073 / 1:100 | Endothelial lineage exclusion |
| PE Rat Anti-Mouse TER-119 | BD Pharmingen™ | 561071 / 1:100 | Erythroid lineage exclusion |
| APC Rat Anti-Mouse CD51 | Elabscience | E-AB-F1235E /1:20 | MSCs positive identification |

Colony-forming unit (CFU) assays were used to functionally assess the proliferative and differentiation potential of hematopoietic progenitors. Although traditionally applied to bone marrow and peripheral blood, this method has also been used to identify hematopoietic progenitors in glioblastoma cell suspensions [4]. CFU assays were performed using the methylcellulose-based medium MethoCult™ M3434 (StemCell Technologies) on three sample types: bone marrow, peripheral blood, and brain tissue (from healthy and stroke-affected mice). Bone marrow was isolated from femur, tibia, and pelvis, crushed in cold PBS, filtered (100 µm), centrifuged (1500 rpm, 5 min, 4°C), and subjected to red blood cell lysis (1:10 buffer dilution). Peripheral blood was collected via cardiac puncture, and red blood cells were lysed following the same protocol as for the bone marrow. The resulting cells were washed, filtered, and resuspended in PBS at 8 × 10⁴ cells/200 µL. Brain tissue was mechanically dissociated, filtered, and centrifuged to obtain single-cell suspensions, which were also resuspended at 8 × 10⁴ cells/200 µL. For each sample, 200 µL of the cell suspension were mixed with 3.8 mL of MethoCult. The mixture was vortexed briefly (5–10 s) and allowed to rest for 15 min under sterile conditions to eliminate air bubbles. From the 4-mL mixture prepared for each sample, 3 mL were used for plating (1 mL per 35-mm grid-marked dish). The plates were placed in humidified chambers at 37°C and incubated for 7–14 days. Colony formation was then assessed based on morphology and lineage-specific criteria, and the mean colony number per initial 200-µL sample was calculated from the three replicates.

**References**

1. Martín-Martín Y, Fernández-García L, Sanchez-Rebato MH, et al. Evaluation of Neurosecretome from Mesenchymal Stem Cells Encapsulated in Silk Fibroin Hydrogels. Sci Rep. 2019;9(1):8801. Published 2019 Jun 19. doi:10.1038/s41598-019-45238-4
2. Dominici M, Le Blanc K, Mueller I, et al. Minimal criteria for defining multipotent mesenchymal stromal cells. The International Society for Cellular Therapy position statement. Cytotherapy. 2006;8(4):315-317. doi:10.1080/14653240600855905.
3. Boulais PE, Mizoguchi T, Zimmerman S, et al. The Majority of CD45- Ter119- CD31- Bone Marrow Cell Fraction Is of Hematopoietic Origin and Contains Erythroid and Lymphoid Progenitors. Immunity. 2018;49(4):627-639.e6. doi:10.1016/j.immuni.2018.08.019.
4. Lu IN, Dobersalske C, Rauschenbach L, et al. Tumor-associated hematopoietic stem and progenitor cells positively linked to glioblastoma progression. Nat Commun. 2021;12(1):3895. Published 2021 Jun 23. doi:10.1038/s41467-021-23995-z


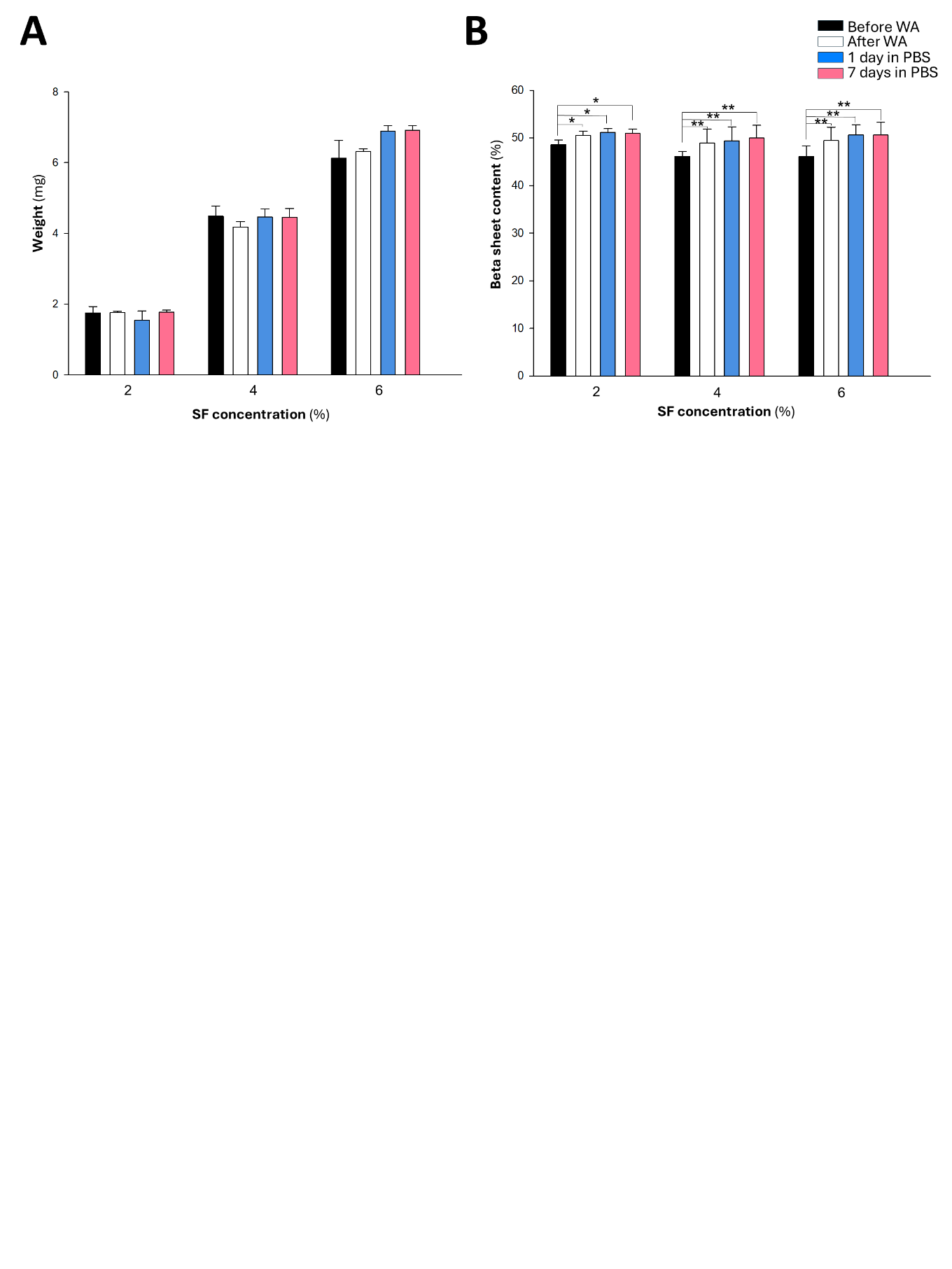


**Supplementary Figure S1. Stability of different SF films concentrations (2%, 4% and 6%) over time in PBS at 37ºC.** (A) Film mass (mg) measured before water annealing (WA), immediately after WA, and WA films after 1 and 7 days of incubation in PBS at 37ºC. WA was performed for 1 h at room temperature in a saturated water-vapor chamber to induce β-sheet formation and insolubilization. (B) β-sheet content (%) quantified by FTIR analysis of the amide I band (≈1600–1700 cm⁻¹) for each condition: before WA (black bars), immediately after WA (white bars), and after 1 (blue bars) and 7 (pink bars) days in in PBS at 37ºC. Data are presented as mean ± SEM (n = 5). Two-way ANOVA (**P* < 0.05; ***P* < 0.01).

**
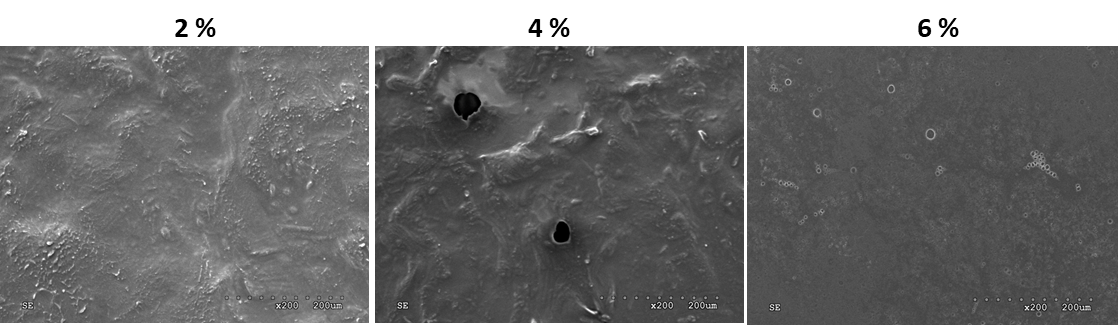
**

**Supplementary Figure S2. Scanning electron microscopy images of silk fibroin films at increasing concentrations.** Surface topography appeared comparable across the tested concentrations. Although isolated defects were observed in the 4% and 6% films, no discernible morphological features suggested a relationship between surface structure and the SDF-1α release profile.


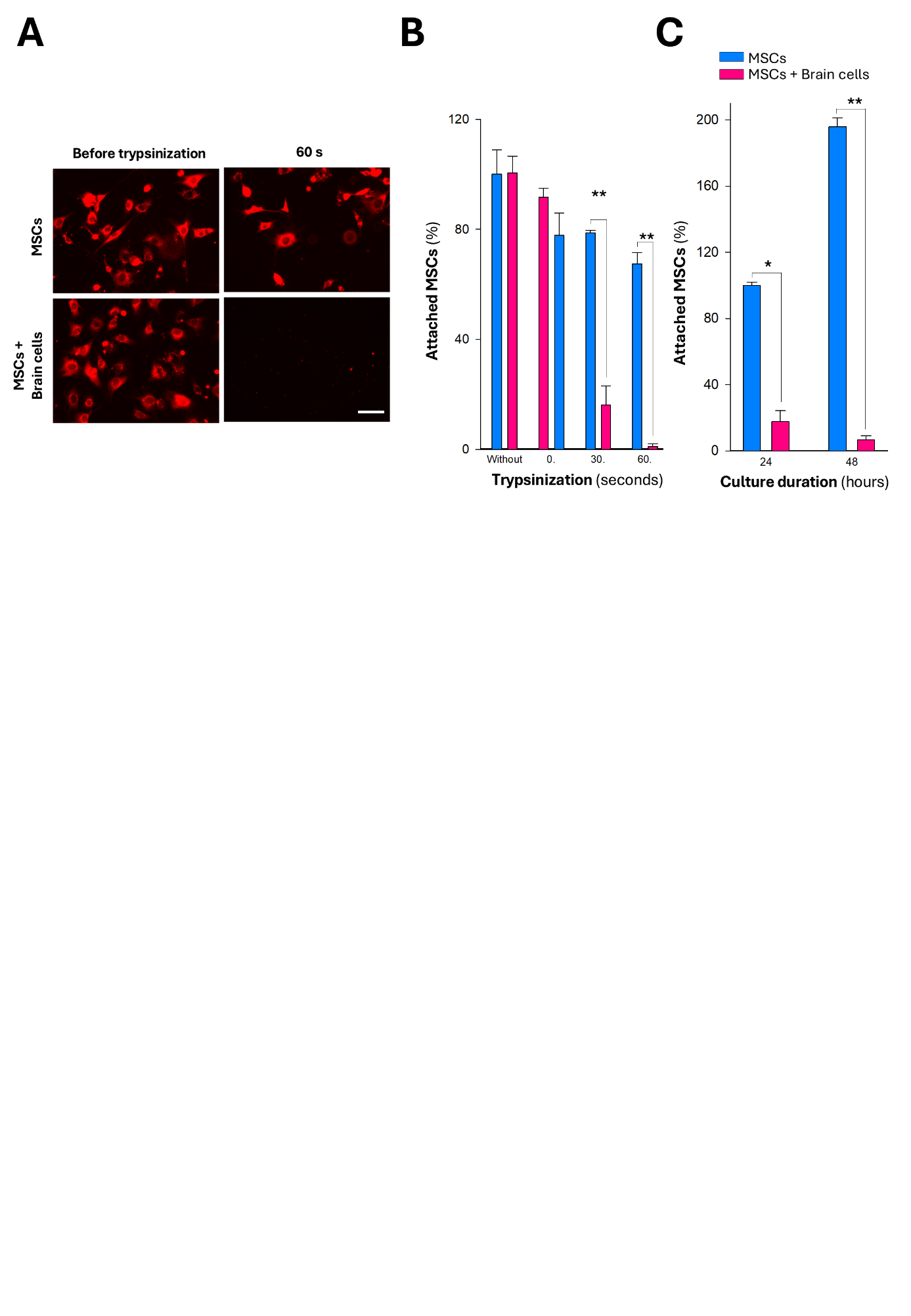


**Supplementary Figure S3. Reduced adhesion and proliferation of MSCs in contact with brain-derived cells.** (A) Representative images of DiI-labeled MSCs (MSCs-Dil) cultured alone or co-cultured with brain-derived cells, before and after trypsinization. This strategy was used to establish a criterion to distinguish highly adherent MSCs from the less adherent brain cells (scale bar, 100 µm). (B) Percentage of MSCs-Dil retained after graded trypsin exposure (0, 30, 60 s; ‘Without’ = no trypsin) in mono-culture (blue bars) or co-culture with brain-derived cells (pink bars). MSCs in co-culture with brain-derived cells are more sensitive to trypsinization and detach more readily, indicating a reduced attachment capacity compared with MSCs cultured alone. (C) Relative proliferation of MSCs. At time zero, equal numbers of MSCs were seeded either alone or in co-culture with brain-derived cells. MSCs in monoculture (blue bars) showed a marked increase in cell number 24 hours after seeding and continued to proliferate over time (48 hours), whereas co-culture with brain-derived cells markedly suppressed their proliferation. This limited proliferative capacity would hinder long-term analyses aimed at achieving a higher enrichment of MSCs relative to brain-derived cells, which would be necessary to clearly distinguish (using the criterion of strong cell adherence) endogenous MSCs from brain cells. Data are presented as mean ± SEM (n = 6). Two-way ANOVA (**P* < 0.05; ***P* < 0.01).


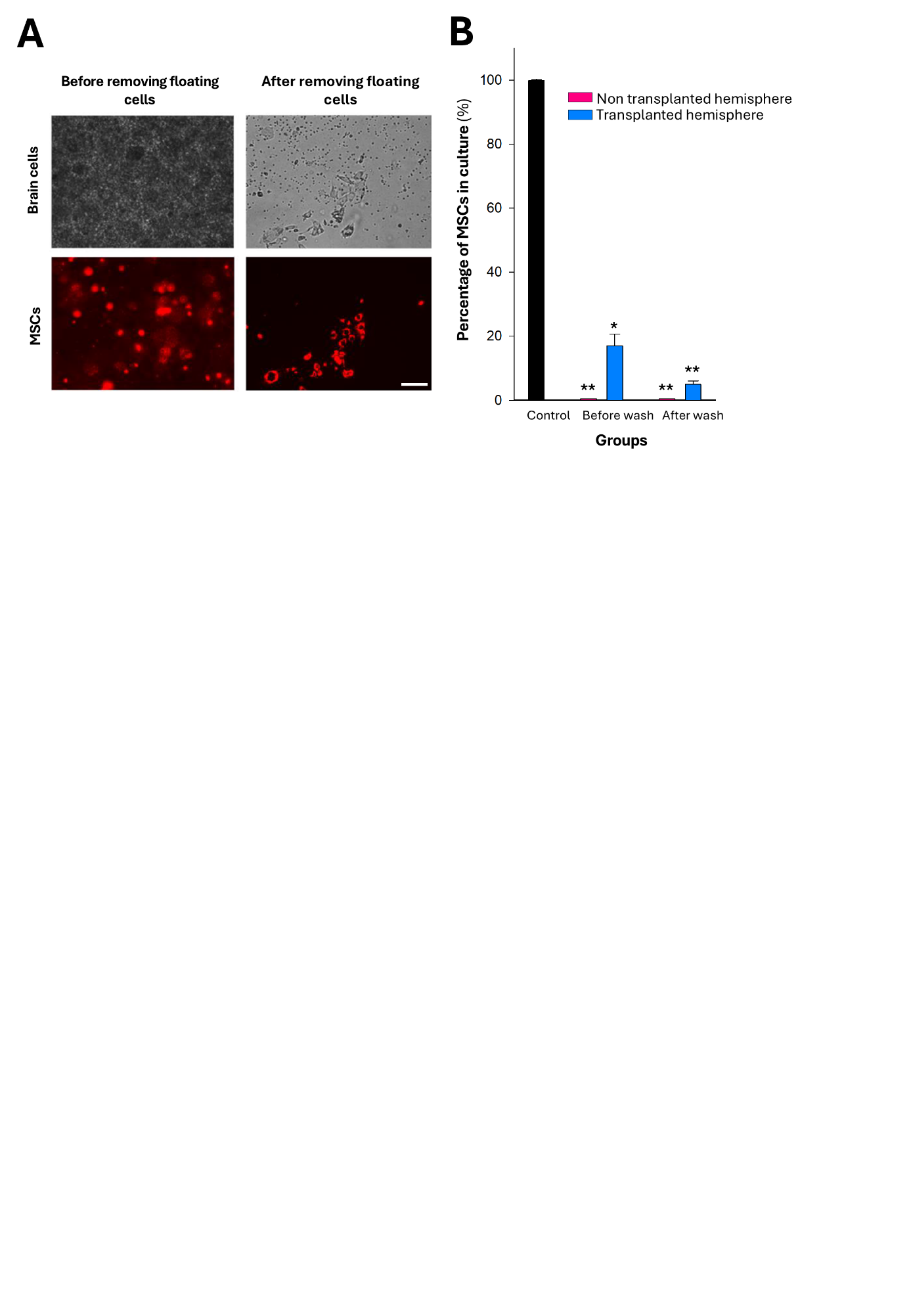


**Supplementary Figure S4. Reduced adhesion of DiI-labeled MSCs after transient *in vivo* exposure to brain tissue.** (A) Representative images of cells recovered from the transplanted and non-transplanted hemispheres 24 hours after intracerebral transplantation of MSCs. After brain tissue dissociation, the recovered cells, containing both DiI-labeled MSCs and brain-derived cells, were cultured for 24 hours to allow cell attachment. Images are shown before and after removal of non-adherent (floating) cells by washing. Red signal identifies DiI-labeled MSCs, and the upper panels show phase/bright-field views of brain-derived cells. After washing, DiI⁺ MSCs are markedly depleted, consistent with their reduced attachment capacity when coexisting with brain-derived cells (scale bar, 100 µm). (B) Quantification of the percentage of MSCs remaining attached after 24 hours of culture following recovery from brain tissue. The same number of MSCs was initially injected or seeded in plastic (input, black bar), allowing comparison of attachment efficiency (output; pink = non-transplanted hemisphere; blue = transplanted hemisphere). Cells recovered from the hemispheres—containing both injected MSCs and brain-derived cells—show a pronounced loss of MSCs, particularly after the wash step, indicating that prior contact with brain tissue reduces both the number and adhesion capacity of MSCs compared with MSCs cultured alone (plastic-only control). Data are presented as mean ± SEM (n = 6). Two-way ANOVA (**P* < 0.05; ***P* < 0.01).


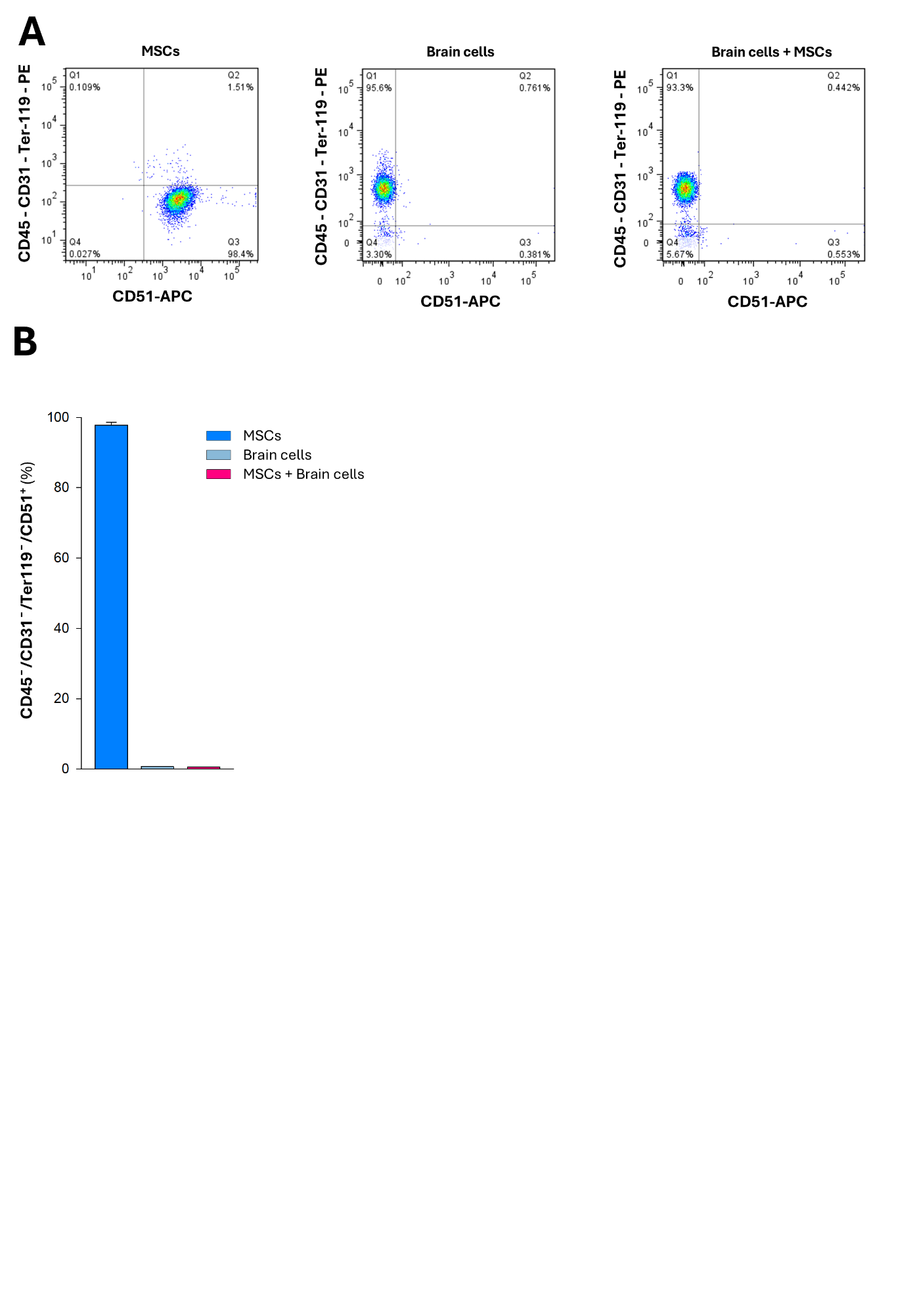


**Supplementary Figure S5. Flow-cytometric detection of MSCs among brain-derived cells.** (A) Representative dot plots of CD51-APC (x-axis) versus a lineage cocktail (CD45-PE/CD31-PE/Ter119-PE, y-axis) for MSCs alone, brain-derived cells, and brain cells + MSCs. The Q3 gate (CD45^-^/CD31^-^/Ter119^-^/APC^+^) was defined a priori to capture the canonical MSCs profile; this gate does not overlap with the distribution of brain-derived cells. In mixed samples, events within Q3 are markedly reduced. (B) Quantification of the Q3 population (%) for MSCs, brain-derived cells, and mixed samples. MSCs are highly represented in Q3 (blue bars), while brain-derived cells contribute negligibly (light blue bars). Following *in vivo* transplantation of MSCs into the brain, Q3 events are nearly absent (pink bars), indicating that these conditions are unfavorable for reliable MSCs detection. Data are presented as mean ± SEM (for MSCs and brain cells n = 6, and for MSCs transplantation in the brain n = 3).


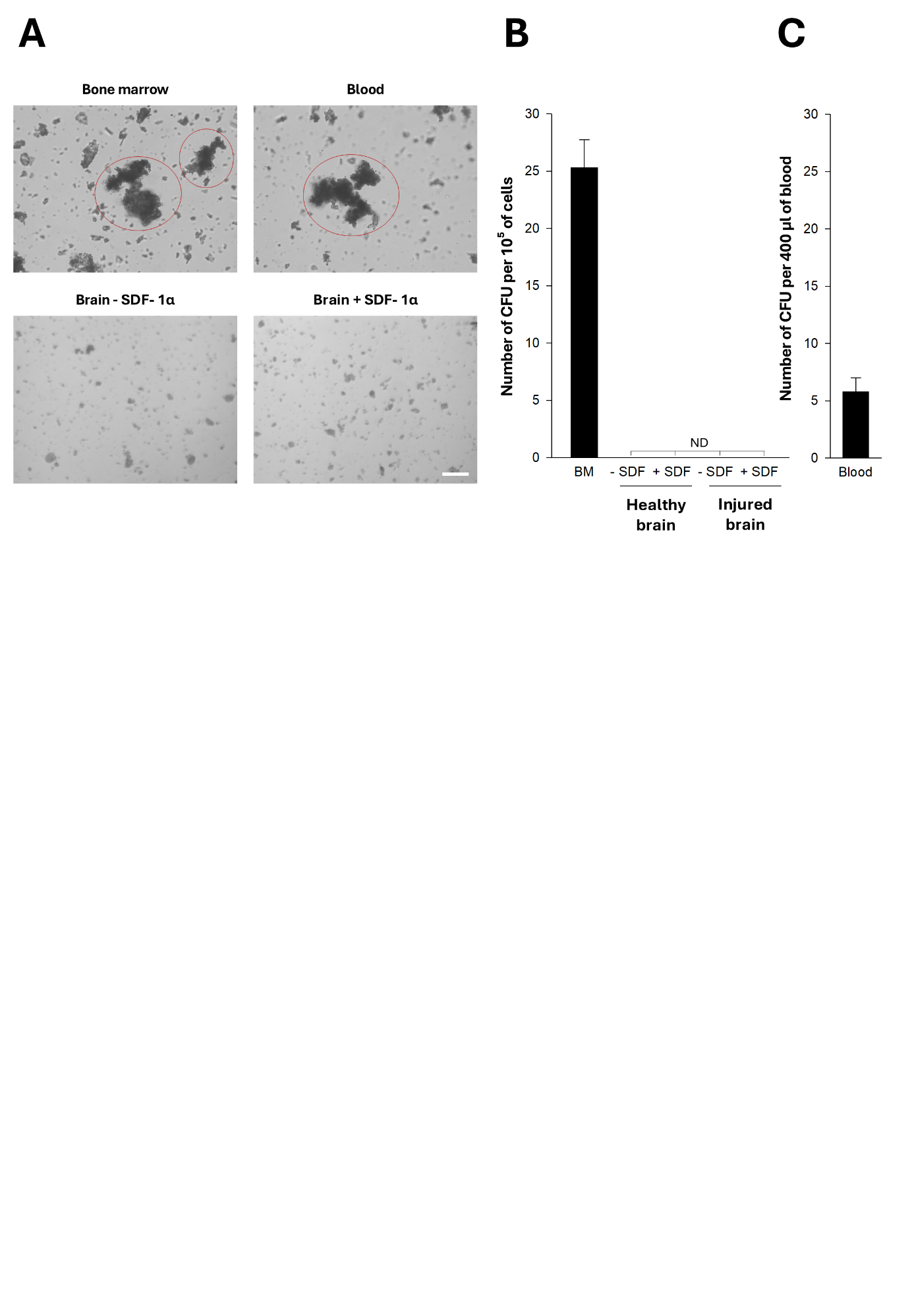


**Supplementary Figure S6. Colony-forming unit (CFU) assay to detect hematopoietic progenitor cells in bone marrow, blood, and brain-derived preparations.** (A) Representative CFU images after 10–14 days in methylcellulose. Robust colonies are evident in bone marrow and peripheral blood controls (red circles), whereas brain-derived cell suspensions—from healthy mice and injured (brain stroke) mice with or without SDF-1α injection—yielded no detectable colonies. Scale bar, 100 µm. (B) Quantification of CFU per 10^5^ cells for bone marrow and brain-derived samples under the indicated conditions; ND = not detected. (C) CFU per 400 µL peripheral blood. CFU assays are the gold standard for functional identification of hematopoietic progenitors; only bone marrow and blood formed colonies, confirming the absence of recoverable hematopoietic progenitors in brain-derived cultures from both healthy and injured tissue, irrespective of SDF-1α. Data are presented as mean ± SEM (for bone marrow and blood n = 5, and for brain n = 3 per condition).


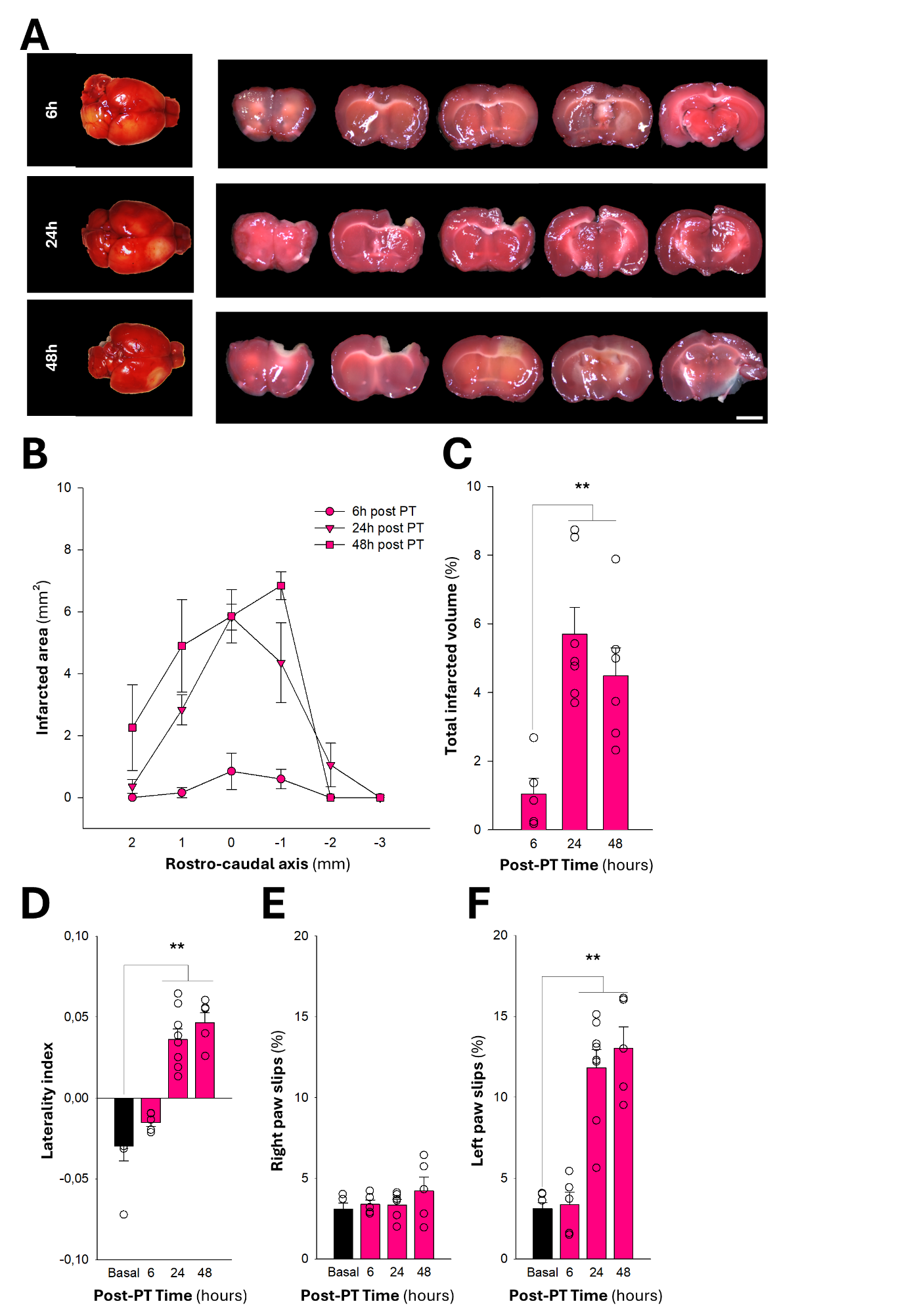


**Supplementary Figure S7. Characterization of the photothrombotic (PT) stroke model targeting primary somatosensory cortex (S1).** (A) Representative TTC-stained coronal sections at 6, 24, and 48 h post-PT show the evolution of infarcted cortex (pale) along the rostrocaudal axis. The infarct core enlarges from 6 to 24 h and then stabilizes, indicating that lesion maturation is largely complete within 24 h. Scale bar, 2 mm. (B) Rostrocaudal profiles of infarct area (mm²) show a significant expansion at 24 h, peaking near bregma 0 mm, with no further increase at 48 h. (C) Total infarct volume (%) is greater at 24 h than at 6 h and does not increase at 48 h, consistent with a plateau in lesion progression. (D–F) Behavioral readouts reveal stable contralateral forelimb deficits from 24 h onward: the cylinder test (laterality index) shows marked sensorimotor asymmetry at 24 and 48 h (D), and grid-walking quantifies paw slips for the right (E) and left (F) forepaws, with the left (contralateral) forepaw exhibiting the highest error rate at 48 h. Data are mean ± SEM (B– C: n = 5–7; D–F: n = 5–8). One-way ANOVA (***P* < 0.01).


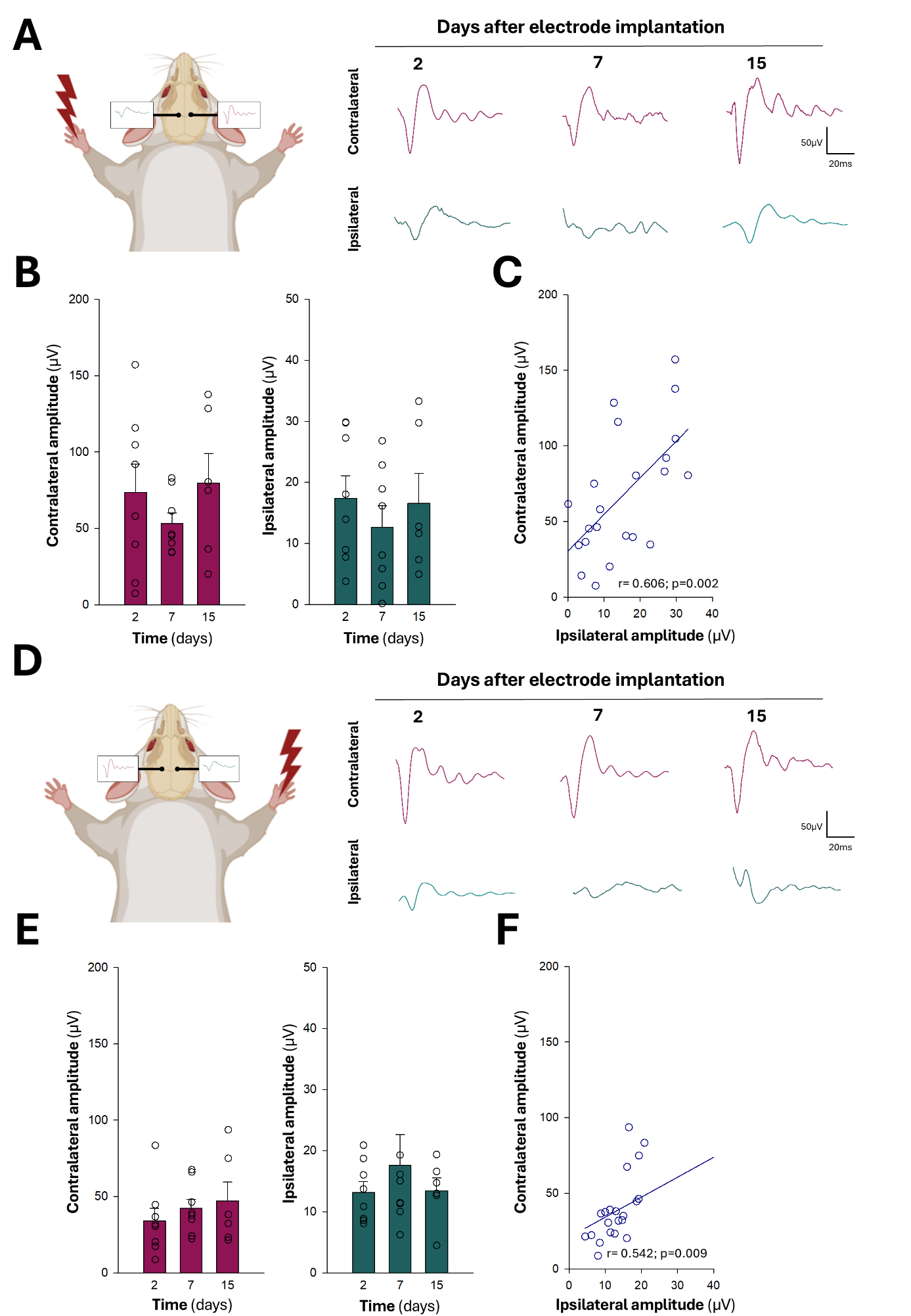


**Supplementary Figure S8.** **Correlation between ipsilateral and contralateral somatosensory evoked potentials (SSEP) in healthy mice.** (A) Schematic of recording montage and representative SSEP traces evoked by left forepaw stimulation at 2, 7, and 15 days after electrode implantation, shown for contralateral and ipsilateral cortices (scale: 50 µV, 20 ms). (B) Quantification of SSEP amplitude recorded contralaterally (right hemisphere) and ipsilaterally (left hemisphere) following left forepaw stimulation. (C) Pearson correlation between ipsilateral and contralateral amplitudes for left-paw stimulation. (D) Schematic and representative SSEP traces evoked by right forepaw stimulation at the same time points (scale: 50 µV, 20 ms). (E) Quantification of SSEP amplitude recorded contralaterally (left hemisphere) and ipsilaterally (right hemisphere) following right forepaw stimulation. (F) Pearson correlation between ipsilateral and contralateral amplitudes for right-paw stimulation. Data are presented as mean ± SEM (n = 6–8 mice).


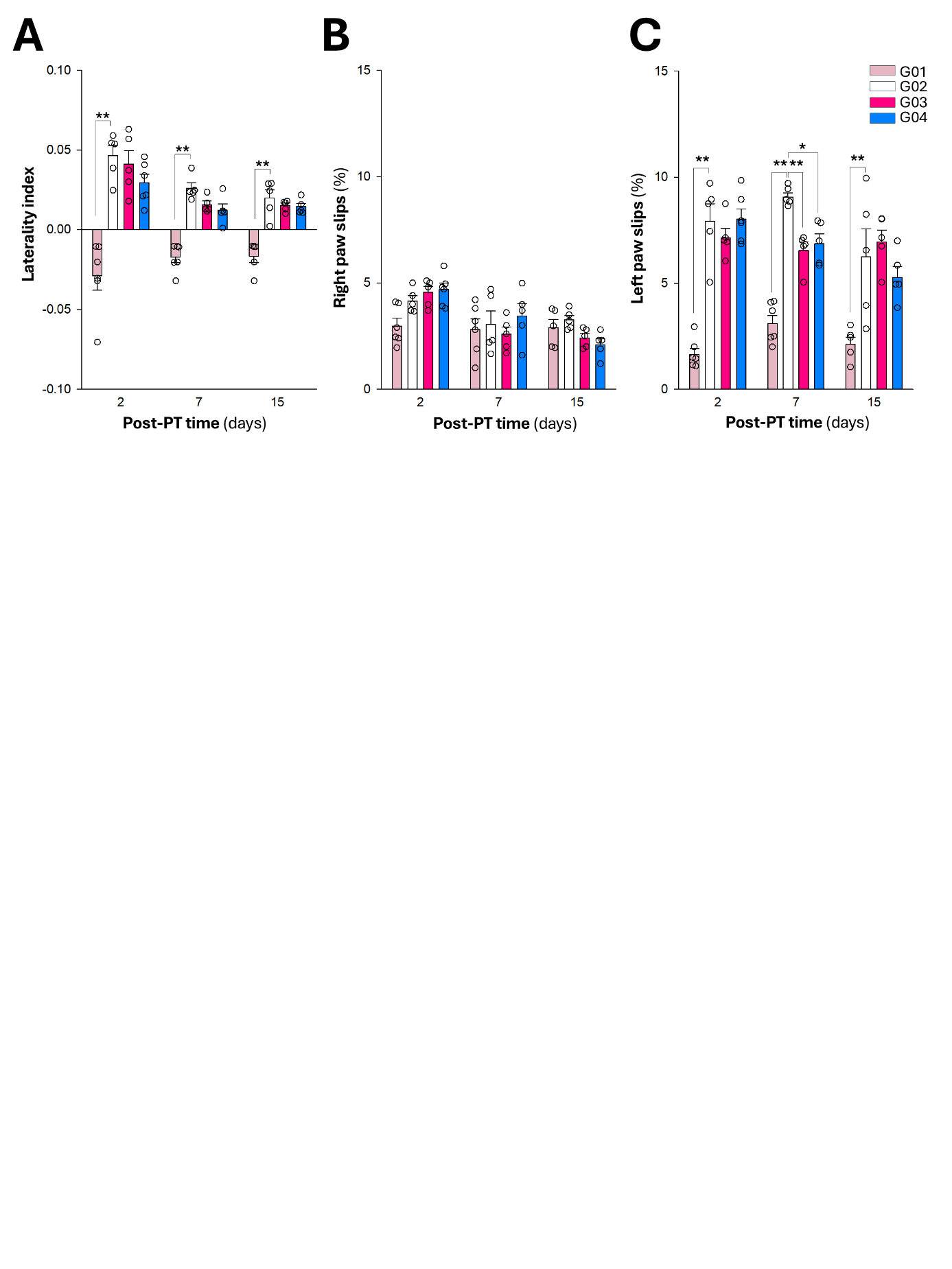


**Supplementary Figure S9.** **Behavioral assessment of motor asymmetry following SDF 1α–SF film treatment in a photothrombotic (PT) stroke model.** (A) Cylinder test evaluating forelimb use asymmetry, expressed as the laterality index, at 2, 7, and 15 days post-PT across four experimental groups: G01 (sham healthy control), G02 (PT + no treatment), G03 (PT + SF film), and G04 (PT + SDF-1α–SF film). Higher positive laterality index values indicate greater impairment in the use of the contralateral limb relative to the infarcted right hemisphere. Grid-walking test results showing right paw slips (B) and left paw slips (C) at the same time points. Increased left paw slips indicate impaired sensorimotor coordination on the side contralateral to the infarct. Data are presented as mean ± SEM (n = 5–6). Two-way ANOVA was performed (**P* < 0.05, ***P* < 0.01); comparisons are versus G02 at the same time point.

.
